# Genetic Diversity and Resistance to Fusarium Head Blight in Synthetic Hexaploid Wheat Derived From *Aegilops tauschii* and Diverse *Triticum turgidum* Subspecies

**DOI:** 10.3389/fpls.2018.01829

**Published:** 2018-12-11

**Authors:** Agnes Szabo-Hever, Qijun Zhang, Timothy L. Friesen, Shaobin Zhong, Elias M. Elias, Xiwen Cai, Yue Jin, Justin D. Faris, Shiaoman Chao, Steven S. Xu

**Affiliations:** ^1^Cereal Crops Research Unit, Edward T. Schafer Agricultural Research Center, Agricultural Research Service, United States Department of Agriculture, Fargo, ND, United States; ^2^Department of Plant Sciences, North Dakota State University, Fargo, ND, United States; ^3^Department of Plant Pathology, North Dakota State University, Fargo, ND, United States; ^4^Cereal Disease Laboratory, Agricultural Research Service, United States Department of Agriculture, St. Paul, MN, United States

**Keywords:** wheat, synthetic hexaploid wheat, *Aegilops tauschii*, tetraploid wheat, *Triticum turgidum*, Fusarium head blight, genetic diversity

## Abstract

Synthetic hexaploid wheat (SHW) can serve as a bridge for the transfer of useful genes from *Aegilops tauschii* and tetraploid wheat (*Triticum turgidum*) into common wheat (*T. aestivum*). The objective of this study was to evaluate 149 SHW lines and their 74 tetraploid parents for their genetic diversity, breeding values and inter-genomic interactions for resistance to Fusarium head blight (FHB). The genetic diversity analysis was performed based on the population structure established using 4,674 and 3,330 polymorphic SNP markers among the SHW lines and tetraploid parents, respectively. The results showed that all *T. carthlicum* and most *T. dicoccum* accessions formed different clusters and subpopulations, respectively, whereas all the *T. durum*, *T. polonicum*, *T. turgidum*, and *T. turanicum* accessions were clustered together, suggesting that *T. durum* was more closely related to *T. polonicum*, *T. turgidum*, and *T. turanicum* than to *T. dicoccum*. The genetic diversity of the SHW lines mainly reflected that of the tetraploid parents. The SHW lines and their tetraploid parents were evaluated for reactions to FHB in two greenhouse seasons and at two field nurseries for 2 years. As expected, most of the SHW lines were more resistant than their tetraploid parents in all environments. The FHB severities of the SHW lines varied greatly depending on the *Ae. tauschii* and tetraploid genotypes involved. Most of the SHW lines with a high level of FHB resistance were generally derived from the tetraploid accessions with a high level of FHB resistance. Among the 149 SHW lines, 140 were developed by using three *Ae. tauschii* accessions CIae 26, PI 268210, and RL 5286. These SHW lines showed FHB severities reduced by 21.7%, 17.3%, and 11.5%, respectively, with an average reduction of 18.3%, as compared to the tetraploid parents, suggesting that the D genome may play a major role in reducing disease severity in the SHW lines. Thirteen SHW lines consistently showed a high level of FHB resistance compared to the resistant check, Sumai 3, in each environment. These SHW lines will be useful for the development of FHB-resistant wheat germplasm and populations for discovery of novel FHB resistance genes.

## Introduction

Fusarium head blight (FHB), also known as scab, is a destructive disease of durum wheat [*Triticum turgidum* L. ssp. *durum* (Desf.) Husn., 2*n* = 4*x* = 28, AABB] and common wheat (*T. aestivum* L. em Thell., 2*n* = 6*x* = 42, AABBDD) in the humid and semi-humid wheat-growing areas of the world (Schroeder and Christensen, 1963). This disease, mainly caused by fungal pathogen *Fusarium graminearum* Schwabe [teleomorph *Gibberella zeae* (Schw.) Petch.] in North America, can lead to severe losses not only in grain yield but also in quality. Mycotoxins, the secondary metabolites of this pathogen, make the harvested grain unsuitable for consumption as food or feed (Gilbert and Tekauz, 2000). Since the early 1990s, FHB has become a serious threat to wheat production globally due to its frequent outbreaks in many wheat-growing regions including the United States, Canada, Europe, and China (see review by McMullen et al., 1997, 2012; Zhang et al., 2012). The severe epidemics of this disease in North America in the early 1990s resulted in an estimated loss of at least 100 million bushels annually for the years 1991, 1993, and 1994 (McMullen et al., 1997). A recent estimate for the value of yield loss for wheat in the United States was $1.176 billion in 2015 and 2016 (Wilson et al., 2018). To confine this threat, an emphasis has been placed on FHB resistance breeding in wheat. Tremendous work had been put into finding new resistance sources with a focus mainly on the resistance present in the exotic wheat germplasm from China and various gene banks. As a result, more than 50 FHB resistance quantitative trait loci (QTL) have been identified, and the most notable QTL were mapped on chromosome arms 3BS (*Fhb1*), 5AS (*Qfhs.ifa-5A*), 5AL (*Qfhb.rwg-5A.2*), and 6BS (*Fhb2*) from common wheat ‘Sumai 3’ and PI 277012 (see reviewed by Buerstmayr et al., 2009; Chu et al., 2011; Zhao et al., 2018b).

To widen the genetic resources of FHB resistance, it is necessary to identify and transfer novel resistance QTL from the germplasms of wheat and its related species that have not been tapped for FHB. Hexaploid wheat is known to originate as a result of hybridization between an AB genome-containing tetraploid wheat (*T. turgidum* spp., 2*n* = 4*x* = 28, AABB) and the diploid goatgrass *Aegilops tauschii* Cosson (2*n* = 2*x* = 14, DD), which contributed the D genome (Kihara, 1944; McFadden and Sears, 1946). Therefore, the world core collections of eight tetraploid wheat subspecies [*T. turgidum* ssp. *carthlicum* (Nevski) Á. Löve & D. Löve, *T. turgidum* ssp. *dicoccoides* (Körn. ex Asch. & Graebner) Thell., *T. turgidum* ssp. *dicoccum* (Schrank ex Schübler) Thell., *T. turgidum* ssp. *durum*, *T. turgidum* ssp. *polonicum* (L.) Thell., *T. turgidum* ssp. *turanicum* (Jakubz.) Á. Löve & D. Löve, *T. turgidum* ssp. *paleocolchicum* (Menabde) Á. Löve & D. Löve, and *T. turgidum* ssp. *turgidum*, which are abbreviated as *T. carthlicum*, *T. dicoccoides*, *T. dicoccum*, *T. durum*, *T. polonicum*, *T. turanicum*, *T. paleocolchicum*, and *T. turgidum*, respectively] and *Ae. tauschii* have been considered as invaluable genetic resources for wheat improvement (Börner et al., 2015; Fedak, 2015; Arora et al., 2018). Many unique genes for resistance to several major wheat diseases and insects, such as rusts, powdery mildew, Hessian fly, and greenbug, have been transferred from tetraploid wheat and/or *Ae. tauschii* into common wheat and extensively used in wheat breeding and production globally (see reviews by Ogbonnaya et al., 2013; Börner et al., 2015; Fedak, 2015).

Tetraploid wheat and *Ae. tauschii* have been used less as sources of FHB resistance because tetraploid wheat is generally more susceptible to FHB than hexaploid wheat, and *Ae. tauschii* plants are not suitable for direct evaluation for FHB resistance in field conditions because of their premature seed shattering nature. Buerstmayr et al. (2012) suggested that tetraploid durum wheat, which is known to be highly susceptible to FHB, does not necessarily lack FHB resistance alleles. Their findings that a resistance QTL introgressed from hexaploid wheat into durum improved resistance in only a few cases supported the hypothesis that either most durum wheat genotypes possess suppressors that silence or reduce the effect of resistance QTL (Stack et al., 2002; Garvin et al., 2009), or the D genome contributes resistance-inducing genes that are absent in durum wheat (Fakhfakh et al., 2011). Actually, a number of accessions of *T. dicoccoides* (Miller et al., 1998; Buerstmayr et al., 2003; Oliver et al., 2007), *T. dicoccum* (Oliver et al., 2008), *T. polonicum* (Wiwart et al., 2013), and *T. carthlicum* (Oliver et al., 2008) were identified to have moderate to high levels of FHB resistance. Several FHB resistance QTL were identified in durum wheat (Somers et al., 2006; Zhang et al., 2014; Zhao et al., 2018a), *T. dicoccoides* (Otto et al., 2002; Stack and Faris, 2006; Chen et al., 2007; Gladysz et al., 2007; Kumar et al., 2007; Buerstmayr et al., 2013), *T. dicoccum* (Buerstmayr et al., 2012; Zhang et al., 2014), and *T. carthlicum* (Somers et al., 2006). Brisco et al. (2017) identified five and seven *Ae. tauschii* accessions showing resistance and moderate resistance, respectively, suggesting that *Ae. tauschii* can be a potential source of novel FHB resistance.

One way to bring potential new resistance genes from tetraploid wheat and *Ae. tauschii* germplasm into wheat breeding programs is the development of synthetic hexaploid wheat (SHW) (*xAegilotriticum* spp., 2*n* = 6*x* = 42, AABBDD). Since the 1940s, over 1,500 SHW lines have been developed and a large number of the SHW lines have been identified to exhibit resistance to major wheat diseases (rusts, Septoria, barley yellow dwarf virus, crown rot, tan spot, spot blotch, nematodes, powdery mildew, FHB, etc.) and insects (Hessian fly and greenbug) and tolerance to abiotic stresses (drought, heat, salinity/sodicity, and waterlogging) as well as novel grain yield and quality traits (see review by Ogbonnaya et al., 2013). A large number of adapted wheat germplasms and populations have been developed from elite SHW lines (Lazar et al., 1996; Yang et al., 2006, 2009; Dreisigacker et al., 2008; Lage and Trethowan, 2008; Jafarzadeh et al., 2016) and some of SHW-derived germplasms have been successfully utilized to develop common wheat varieties, such as the highly yielding variety ‘Chuanmai 42’ (Yang et al., 2009) and greenbug-resistant varieties ‘TAM 110’ (Lazar et al., 1997) and ‘TAM 112’ (Rudd et al., 2014). Previous efforts to develop SHW germplasm for wheat improvement have mostly targeted the genetic diversity of the D genome present in world core collections of *Ae. tauschii*. Noticeably, most of the SHW lines that are currently available were developed from the crosses between durum wheat and diverse *Ae. tauschii* accessions by L. R. Joppa at USDA-ARS (Fargo, ND, United States; Xu et al., 2010) and Mujeeb-Kazi and Delgado (2001) and Mujeeb-Kazi (2003) at the International Maize and Wheat Improvement Center, Mexico (CIMMYT). Therefore, most of the tetraploid wheat germplasm resources other than durum have not been intentionally utilized for SHW production for wheat breeding programs except for a small number of *T. dicoccoides*, *T. carthlicum*, and *T. dicoccum* accessions that were sporadically used (Lange and Jochemsen, 1992; Xu and Dong, 1992; Lage et al., 2006).

To incorporate the genetic diversity from under-exploited tetraploids into SHW germplasm resource, we recently developed 200 new SHW lines, with 178 lines being developed using six tetraploid subspecies *T. carthlicum*, *T. dicoccum*, *T. dicoccoides*, *T. polonicum*, *T. turgidum*, and *T. turanicum*. These new SHW lines plus durum Langdon-derived SHW lines previously developed by L. R. Joppa (Xu et al., 2010) represent a unique resource for wheat improvement and for investigating polyploidization and intergenomic interactions in wheat. The objectives of this study were to identify FHB resistant SHW lines and to investigate the effect of the D-genome chromosomes derived from various *Ae. tauschii* accessions on FHB resistance by evaluating the genetic diversity and FHB resistance in a subset of 149 SHW lines and their 74 tetraploid parents.

## Materials and Methods

### Plant Materials

A total of 149 SHW lines and their tetraploid wheat (*T. turgidum* L.) parents were used in genetic diversity analysis and evaluation for resistance to FHB. These SHW lines were developed from crossing 10 *Ae. tauschii* accessions to 74 tetraploid wheat accessions belonging to durum wheat and five other tetraploid wheat subspecies (*T*. *carthlicum*, *T*. *dicoccum*, *T*. *polonicum*, *T*. *turgidum*, and *T*. *turanicum*). The accession or line numbers and sources of the tetraploid wheat and *Ae. tauschii* accessions are listed in Supplementary Table S1, and the line numbers and pedigrees of the SHW lines are listed in Supplementary Table S2. Of the 10 *Ae. tauschii* accessions, four (CIae 17, PI 268210, RL 5286, and TA 2377) and six (CIae 19, CIae 22, CIae 25, CIae 26, PI 476874, and TA 1675) were classified as subspecies *strangulata* and *tauschii*, respectively. Three (CIae 26, PI 268210, and RL 5286) of the *Ae. tauschii* accessions were used as the parents of 140 (94%) SHW lines. Except for seven durum ‘Langdon’-derived SHW lines (SW7, SW8, SW9, SW25, SW52, SW53, and SW59) developed by Dr. L. R. Joppa (Xu et al., 2010), all other lines were recently produced by crossing seven *Ae. tauschii* accessions (CI 22, CIae 26, PI 268210, RL 5286, PI 476874, TA 1675, TA 2377) with the 74 tetraploid wheat accessions.

### Genetic Diversity Analysis on the SHW Lines and Their Tetraploid Wheat Parents

The SHW lines and their tetraploid parents were genotyped with the Illumina iSelect wheat 9K array containing 9,000 gene-derived SNPs (Cavanagh et al., 2013) using Illumina’s Infinium method following the manufacturer’s protocols (Illumina Inc., San Diego, CA, United States). The SNP genotype calls were performed using the genotyping module implemented in the Illumina’s GenomeStudio software v.2011.1. Genotype data were manually inspected for call accuracy before exporting the SNP data file. Heterozygote calls were converted into missing data, markers with poorly separated clusters were excluded, and SNPs with a missing data rate of 10% or higher as well as those with minor allele frequency (MAF) of 0.05 or lower were filtered out. The high-density SNP-based consensus map developed by Maccaferri et al. (2015) for tetraploid wheat and the consensus map previously produced by Cavanagh et al. (2013) for hexaploid wheat were used to remove SNPs with no map information. The final SNP data set for molecular analysis consisted of 3,330 and 4,674 markers for tetraploid parents and SHW lines, respectively.

Polymorphic information content (PIC) was applied to assess genetic diversity and was calculated for single loci as

$$PIC=1-\sum_{\text{i}=1}^{k}P_{\text{i}}^{2}$$

where *k* is the total number of alleles detected for a given marker locus and *P_i_* is the frequency of the *i*-th allele in the set of genotypes investigated (Anderson et al., 1993). In our analysis the PIC = 1 - (*p*^2^+ *q*^2^) formula was used, where *p* and *q* denote the frequencies of the two alleles (Ghislain et al., 1999).

Genetic diversity present among SHW lines and their tetraploid parents was evaluated using both principle component analysis (PCA) in TASSEL4 (Bradbury et al., 2007) and cluster analysis in the R program^1^.

### FHB Resistance Evaluation

Evaluation experiments were performed for evaluating Type II resistance (resistance to spread in the spike) in both greenhouse condition and field nurseries based on the well-established procedures for plant culture, inoculation, and disease scoring as described by Chu et al. (2011) and Zhang et al. (2014). Out of the 74 tetraploid parents, only one accession (*T. dicoccum* PI 272572) was not evaluated because of the low germination rate. In the field and greenhouse evaluation experiments, common wheat varieties ‘Sumai 3’ and ‘Grandin’ were used as resistant and susceptible checks, respectively. In greenhouse experiments, a total of 224 genotypes (149 SHW lines, 73 tetraploid parents, and two checks) were evaluated in two seasons in winter 2015 and 2016, respectively, using a randomized complete block design (RCBD) with three replicates. Each genotype was planted in a plastic pot (16.2 × 18.4 cm) with four seeds for each genotype per replicate. Therefore, a total of 12 plants per genotype were evaluated for most of the lines in each greenhouse experiment. The greenhouse settings for photoperiod and temperature were 16 h and 22°C, respectively. The inoculum was prepared at a concentration of 50,000 spores mL^-1^ from three strains of pathogenic *F*. *graminearum*. For inoculation, 10 μL of inoculum was injected into a single central spikelet near the center of each spike at anthesis as described by Stack et al. (2002). Each inoculated spike was misted and then covered with a misted plastic bag for 72 h. For each genotype, about 10 spikes in each replicate were inoculated. Disease scoring was performed by counting infected spikelets and total spikelets on each spike at 21 days post-inoculation, and disease severity for each line was calculated as the percentage of total infected spikelets in total spikelets from all the scored spikes.

In the field experiments, the plant materials were planted in mist-irrigated nurseries using a RCBD with three replicates at two locations (Fargo and Prosper, ND, United States) in the summers of 2015 and 2016. Each genotype was planted in a hill plot with 15 seeds. Inoculum was prepared using the grain spawn inoculation method, in which autoclaved corn seeds were infected with a mixture of spores produced separately from 20 *F*. *graminearum* strains, including 10 3ADON (3-acetyl-deoxynivalenol) producers and 10 15ADON (15-acetyl-deoxynivalenol) producers, collected from the field in North Dakota (Puri and Zhong, 2010). At the boot stage of the earliest lines, inoculum was evenly applied among plots at a rate of 35.6 g m^-2^. The nursery was misted for 2 min in 1-h intervals for 12 h daily (4:00 p.m. to 4:00 a.m.), until about 14 days after anthesis of most the genotypes. Ten spikes for each line were individually examined at 21 days post anthesis as the number of infected spikelets per spike using a visual scale: 0 = no spikelets infected, 100 = all spikelets infected based on the method of Stack and McMullen (1998).

Plant height (PH) data were collected in the field experiments, and days to flowering (DTF) data were collected in the field and greenhouse experiments to determine the correlation of these traits with FHB disease severity. PH was measured from the ground surface to the top of the spike excluding the awns. DTF was calculated from January 1 in the greenhouse experiments, and in the field experiments it was calculated from July 1 in 2015 and from June 20 in 2016 when 50% of spikes in a hill were flowering.

### Statistical Analyses

All the statistical analyses were performed separately using evaluation data from hexaploid entries (SHW lines and checks), the tetraploid wheat parents, and the two groups combined. Descriptive statistics were calculated using the software JMP Genomics 7 (SAS Institute, Cary, NC, United States). A normality test for distribution of disease severity was performed using Shapiro-Wilk under the “Goodness of Fit” option using the same program. Bartlett’s test under the general linear model (GLM) procedure was used to test homogeneity of disease severity variances among the experiments using SAS program version 9.4 (SAS Institute). The reduction in FHB severity in the SHW lines was calculated as the difference in FHB severity between the SHW lines and their respective tetraploid parents. To determine the significance of the reduction, the least significant difference (LSD) value was used. Correlation coefficients between disease severity and PH or DTF were calculated using the PROC CORR procedure (SAS Institute). The same procedure was used to calculate the correlation coefficients between the disease severity of SHW lines and their tetraploid parents. To test homogeneity of PH and DTF data, Levene’s test was used under the GLM procedure (SAS Institute). Broad sense heritability was estimated across environments according to Nyquist (1991) with the following formula: H^2^ across environments = 1 - (MS_G__×__E_/MS_G_), where MS_G__×__E_ was mean square genotype × environment; and MS_G_ was mean square genotype.

## Results

### Genetic Diversity of the SHW Lines and Their Tetraploid Wheat Parents

A total of 4,674 SNP markers that were polymorphic among the SHW lines were mapped based on the consensus map previously produced by Cavanagh et al. (2013) resulting in an average of 223 markers per chromosome. The markers spanned a genetic distance of 3,445 cM with an average density of 0.7 cM per marker. However, the average marker density for the D genome was poor at 24 markers per chromosome. The number of markers ranged between 3 (chromosome 4D) and 504 (chromosome 2B). The PIC value was between 0.1 and 0.5 with an average of 0.38 (data not shown). The consensus map developed by Maccaferri et al. (2015) was used to assign the map positions for 3,330 SNP markers for the tetraploid parents resulting in an average of 238 markers per chromosome. The total genetic distance was 2,532.8 cM with an average density of 0.8 cM per marker. The number of markers ranged between 95 (chromosome 4B) and 386 (chromosome 2B). The PIC value varied between 0.1 and 0.5 with an average of 0.39 (data not shown).

**FIGURE 1:**
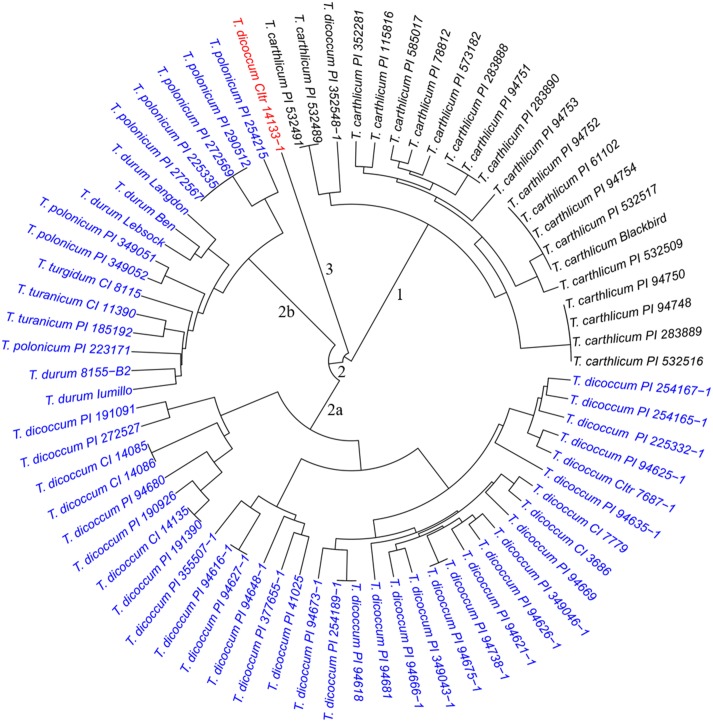
Dendrogram of the 73 tetraploid parents, forming three major clusters. Cluster 1 incluides one *T*. *dicoccum* and 21 *T*. *cathlicum* accesions. Cluster 2a consists of 34 *T*. *dicoccum* accesions. Cluster 2b contains all durum, *T*. *polonicum*, *T*. *turgidum*, and *T*. *turanicum* accessions. Accession *T*. *dicoccum* CItr 14133-1 is an outlier.

**FIGURE 2:**
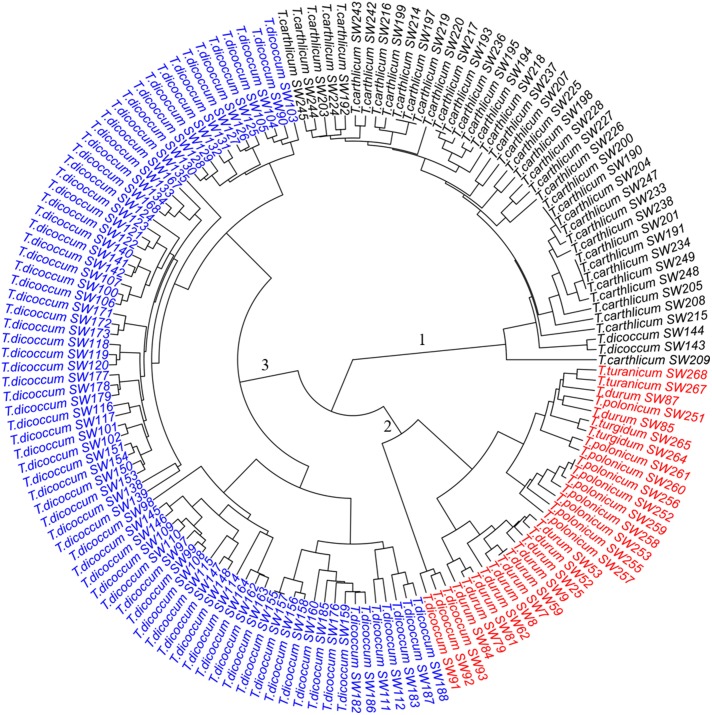
Dendrogram of the 149 SHW lines, forming three major clusters. Cluster 1 consists of 43 SHW lines derived from the accessions in the tetraploid wheat cluster 1. Cluster 2 contains 30 SHW lines corresponding to tetraploid wheat cluster 2b and the outlier *T*. *dicoccum* CItr 14133-1. Cluster 3 contains 76 SHW lines belonging to the tetraploid wheat cluster 2a.

To evaluate genetic similarity, results from PCA indicated that three subpopulations were likely present in both the SHW lines and the tetraploid parents. Results from cluster analysis further confirmed the presence of three major clusters separating 73 tetraploid parents (Figure 1) and 149 SHW lines (Figure 2). The cluster 1 in tetraploid samples (Figure 1, black) consisted of one *T. dicoccum* accession (PI 352548-1) and all 21 *T. carthlicum* accessions. Cluster 2 consisted of 50 individuals (Figure 1, blue) which were further grouped into two subpopulations with one consisting of only *T. dicoccum* accessions (Figure 1, subpopulation 2a) and another containing all durum, *T. polonicum*, *T. turgidum*, and *T. turanicum* accessions (Figure 1, subpopulation 2b). Interestingly, *T. dicoccum* accession CItr 14133-1 (Entry 21) may be an outlier, as it did not share similarity with any of the tetraploid lines (Figure 1, red).

The three clusters separating 149 SHW lines (Figure 2) generally corresponded well with the clustering of the tetraploid parents (Figure 1). Cluster 1 (Figure 2, black) consisted of the 43 SHW lines derived from all the accessions in the tetraploid wheat cluster 1. Cluster 2 (Figure 2, red) contained 30 SHW lines mainly derived from the accessions belonging to tetraploid wheat cluster 2b. Cluster 3 (Figure 2, blue) consisted of 76 SHW lines derived from all the *T. dicoccum* accessions belonging to tetraploid wheat cluster 2a. Although *T. dicoccum* CItr 14133-1 was separated alone from all other tetraploid parents, its three SHW lines (SW91, SW92, and SW93) were grouped into the SHW cluster 2 (Figure 2, red) with the SHW lines derived from tetraploid wheat cluster 2b. These analyses showed that the genetic diversity of this set of SHW lines obviously reflected that of the tetraploid wheat accessions. The results also clearly indicated that among the tetraploid accessions used, including *T. dicoccum*, *T. turgidum*, *T. turanicum*, *T. durum*, and *T. polonicum* lines, *T. durum* was genetically more similar with *T. polonicum* than with *T. dicoccum*.

### Reactions to FHB of SHW Lines and Their Tetraploid Wheat Parents

The 149 SHW lines and their 73 tetraploid parents, together with the two hexaploid checks (Sumai 3 and Grandin), were evaluated for reactions to FHB in two greenhouse seasons and two field nurseries (Fargo and Prosper) in 2 years (Supplementary Table S2). However, two SHW lines, SW9 and SW52 (Entries 4 and 6), and *T. polonicum* accession PI 272567 (Entry 204) were not evaluated in the field experiments in 2015 due to low germination rate. The Bartlett’s test for disease severity variances showed heterogeneity across the two greenhouse seasons and the 2 years of field experiment at two locations (χ^2^_df__=__5_ = 205.6, *P* < 0.0001). However, the data from the field tests showed homogeneity between the two locations in each year (2015: χ^2^_df__=__1_ = 2.88, *P* = 0.0896; 2016: χ^2^_df__=__1_ = 2.31, *P* = 0.1284), as well as between the two greenhouse experiments (χ^2^_df__=__1_ = 2.88, *P* = 0.0895). Therefore, the disease severity data from the two greenhouse experiments (FHBGH) as well as from the field experiments within each year (FHB15 and FHB16) were combined. Thus, these three sets of FHB severity data were used in the subsequent statistical analyses. In addition, the overall mean of disease severity from all the experiments is presented in Supplementary Table S2 to provide general information about the resistance level of each genotype.

**FIGURE 3:**
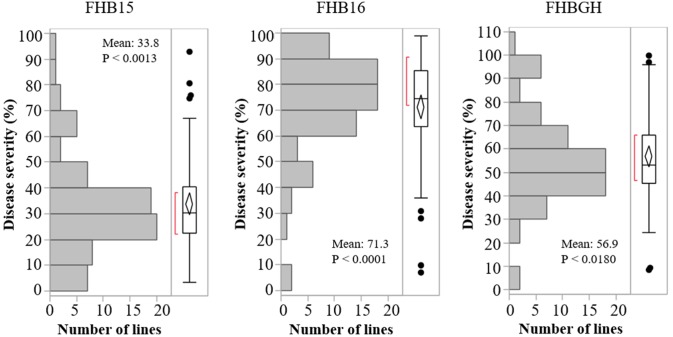
Distribution of Fusarium head blight (FHB) disease severities among the tetraploid wheat lines in the three environments (FHB15, FHB16, and FHBGH). Letter “P” represents probability from normality test for distribution of disease severity.

**FIGURE 4:**
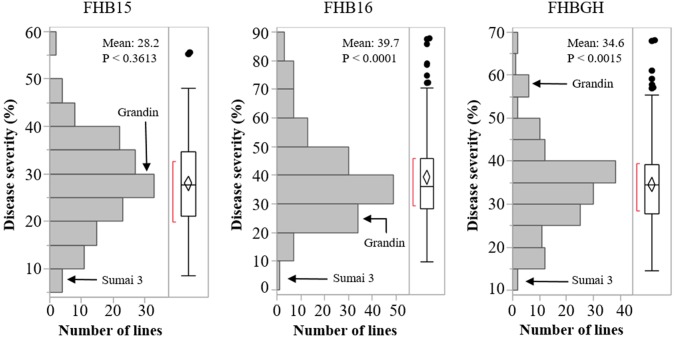
Distribution of Fusarium head blight (FHB) disease severities among the SHW lines in the three environments (FHB15, FHB16, and FHBGH). The two checks, Sumai 3 and Grandin, were included in the data set. Letter “P” represents probability from normality test for distribution of disease severity.

The resistant check Sumai 3 had the expected level of FHB resistance in all environments (FHB severity: 8.6–17.1%) (Table 1 and Supplementary Figure S1). The susceptible check Grandin had the expected level of susceptibility only in the greenhouse experiments (55.3%), but it did not exhibit the expected level of susceptibility in the field environments (26.4% in FHB15 and 24.7% in FHB16) (Table 1 and Supplementary Figure S1). Such low FHB severities were probably caused by early flowering dates of Grandin plants. We observed that Grandin was always among a few lines that had the earliest flowering dates, and it flowered at 9.9, 6.2, 14.7, and 12.4 days earlier than the population average in Prosper and Fargo nurseries in 2015 and 2016, respectively. At the early stage of the experiments, the inoculum pressure was likely not adequately built up. The FHB severity of the SHW lines and tetraploid parents, as expected, was highly variable among different environments. The average FHB severities of the entire population (SHW lines, tetraploid parents, and checks) were 29.9%, 49.8%, and 41.9% in FHB15, FHB16, and FHBGH, respectively (Table 1 and Supplementary Figure S1). The tetraploid parents (average FHB severities: FHB15 = 33.8%, FHB16 = 71.3%, FHBGH = 56.9%) had more variable expressions of FHB than SHW lines (FHB15 = 28.2%, FHB16 = 39.7%, FHBGH = 34.6%) in different experiments (Figures 3, 4). For FHB severity, the heritability (H^2^) values were 0.70, 0.85 and 0.64 among experiments in FHB15, FHB16 and FHBGH, respectively (Table 1), indicating good reproducibility of the experiments.

**Table 1:** Statistical analysis on Fusarium head blight (FHB) severity, days to flowering, and plant height data of a panel of 149 synthetic hexaploid wheat (SHW) lines and their tetraploid wheat parents evaluated in the field and greenhouse experiments.

Data set	Mean	SD	Median	Range	Sumai 3	Grandin	LSD	H^2^
FHB15	29.9	13.5	29.0	3.4–92.8	8.6	26.4	15.8	0.70
FHB16	49.8	22.6	43.5	7.1–98.8	9.7	24.7	19.4	0.85
FHBGH	41.9	17.5	38.1	8.5–100.0	17.1	55.3	13.7	0.64
PH	89.7	8.5	89.5	67.5–130.0	85.4	80.4	5.8	0.90
DTF GH	31.2	6.6	30.8	18.3–60.5	24.8	18.3	4.7	0.86
DTF15 Pro	23.2	5.8	22.0	13.0–37.0	19.7	13.3	4.2	
DTF15 Far	11.9	4.9	11.0	3.0–29.0	5.0	5.7	5.0	
DTF16 Pro	25.7	6.8	24.0	11.0–45.0	24.7	11.0	4.6	
DTF16 Far	25.1	7.5	24.0	10.0–45.0	11.3	12.7	14.7	

*Data set: FHB15 and FHB16 are combined FHB severity data from to field locations (Fargo, Prosper) in 2015 and 2016, respectively; FHBGH is combined FHB severity data from the two greenhouse experiments; PH is combined plant height data from all field experiments (2015, 2016); DTF GH is combined days to flowering data from the two greenhouse experiments; DTF15 Pro and DTF16 Pro are days to flowering data in Prosper in 2015 and 2016, respectively; DTF15 Far and DTF16 Far are days to flowering data in Fargo in 2015 and 2016, respectively. SD, standard deviation; LSD, least significant differences (α < 0.05); H^2^, broad-sense heritability.*

Several resistant genotypes were identified in the field as well as in the greenhouse. Among the 25 most FHB-resistant SHW lines listed in Table 2, 13 lines (SW53, SW87, SW91, SW92, SW93, SW157, SW159, SW162, SW188, SW203, SW252, SW253, and SW261) showed a high level of FHB resistance. Their FHB severities were not significantly different (*P* = 0.05) from Sumai 3 in all three environments in which they were successfully evaluated. Among these SHW lines, eight (61.5%) were derived from the crosses involving *Ae. tauschii* CIae 26, suggesting that *Ae. tauschii* CIae 26 may carry FHB resistance QTL. Three resistant SHW lines SW91, SW92, and SW93 were derived from *T. dicoccum* CItr 14133-1 crossed with three *Ae. tauschii* accessions, indicating that CItr 14133-1 may carry FHB resistance QTL. In fact, CItr 14133-1 showed a high level of FHB resistance in the field conditions with 8.1% and 7.1% disease severities in FHB15 and FHB16, respectively. Similarly, two *T. dicoccum* accessions PI 191091 and *T. dicoccum* PI 272527 showed a high level of resistance comparable to Sumai 3 in all the environments. In particular, PI 272527 had the highest level of FHB resistance among all the tetraploid accessions and SHW lines evaluated in this study, with disease severities being 3.4%, 9.8%, and 8.5% in FHB15, FHB16, and FHBGH, respectively. The two SHW lines SW187 and SW188 derived from PI 272527 also had low disease severities.

**Table 2:** Fusarium head blight (FHB) severity of most resistant synthetic hexaploid wheat (SHW) lines and tetraploid wheat lines.

Entry No.	Line	Materials/Pedigree	Average FHB severity			
			2015	2016	GH	Overall
23	SW92	CItr 14133-1/RL 5286	12.3	10.3	17.1^***^	13.4
22	SW91	CItr 14133-1/CIae 26	9.9	20.0	14.7^***^	14.9
24	SW93	CItr 14133-1/PI 268210	10.5	25.3	14.6^***^	16.8
4	SW9	Langdon/CIae 26	n.d	15.0^***^	19.9^***^	18.3^***^
134	SW187	PI 272527/CIae 26	9.5	29.4^*^	17.8	19.6^**^
216	SW261	PI 349052/CIae 26	15.0^*^	27.7^***^	18.0^***^	20.1^***^
7	SW53	Langdon/PI 268210	11.4	18.6^***^	25.5^***^	20.1^***^
110	SW162	PI 41025/CIae 26	21.3^***^	23.5^***^	16.2^*^	20.3^***^
127	SW182	PI 190926/CIae 26	13.6	33.2	17.1^**^	20.8^*^
203	SW253	PI 254215/CIae 26	19.6^***^	20.0^***^	23.0^***^	20.9^***^
131	SW185	PI 191390/CIae 26	15.5	35.1	19.7^**^	21.5
132	SW186	PI 191390/PI 268210	15.4	32.6	20.9^**^	21.8
104	SW157	CI 14086/CIae 26	20.9	18.5^***^	25.8^***^	21.9^***^
107	SW159	CI 14135/CIae 26	21.5	23.3	21.5	22.1
154	SW203	PI 94753/PI 268210	10.0	27.6^*^	30.5	22.4^*^
102	SW156	CI 14085/PI 268210	25.6	18.8^***^	23.4^***^	22.6^***^
16	SW87	8155-B2/CIae 26	20.3^***^	20.8^***^	25.9^***^	22.7^***^
129	SW183	PI 191091/CIae 26	13.1	38.7	15.2	22.9
223	SW268	PI 185192/PI 268210	14.5^***^	44.0^***^	18.2^***^	23.3^***^
111	SW163	PI 41025/PI 268210	19.9^***^	32.6^***^	19.7	24.5^***^
86	SW143	PI 352548-1/CIae 26	26.6^**^	25.4	22.7^*^	24.9^**^
105	SW158	CI 14086/PI 268210	25.3	23.0^***^	26.6^***^	24.9^***^
201	SW252	PI 225335/CIae 26	15.8^***^	28.3^***^	30.2^***^	25.3^***^
18	SW85	Iumillo/CIae 26	25.2	25.6^***^	25.2^***^	25.3^***^
135	SW188	PI 272527/RL 5286	20.5^*^	26.5	30.3^**^	25.7^***^
133	PI 272527	*T. dicoccum* PI 272527	3.4	9.8	8.5	7.2
21	CItr 14133-1	*T. dicoccum* CItr 14133-1	8.1	7.1	42.5	20.6
128	PI 191091	*T. dicoccum* PI 191091	22.5	28.1	9.3	22.0
106	CI 14135	*T. dicoccum* CI 14135	8.5	36.0	24.4	22.9
130	PI 191390	*T. dicoccum* PI 191390	4.9	41.3	40.3	28.2
	Sumai 3	*T. aestivum*	8.6	9.7	17.1	11.8
	Grandin	*T. aestivum*	26.4	24.7	55.3	35.5

*^∗^, ^∗∗^, and ^∗∗∗^ indicate that the SHW lines were significantly different from their respective tetraploid parents at the 0.05, 0.01, and 0.001 probability levels, respectively (LSD test). Line number followed by “-1” (e.g., CItr 14133-1) indicated a single plant selection from the original seed stock.*

### Effects of Plant Height and Days to Flowering on FHB Severity of SHW Lines and Their Tetraploid Parents

The Levene’s test for PH showed homogeneity of error variances across the field experiments (*P* < 0.1437, *df* = 3), therefore all the experiments were combined for further analyses. The tetraploid parents showed a wide variation in PH, ranging from 67.5 to 130.0 cm, whereas the SHW lines ranged from 73.8 to 108.9 cm (Table 1 and Supplementary Table S3). The DTF data from the greenhouse experiments were combined based on the homogeneity test result (*P* < 0.3242, *df* = 1), whereas the DTF data from field experiments were heterogeneous (*P* < 0.0001, *df* = 3) and were analyzed separately in the further analyses. The plants started flowering early in 2016 (30th of June in Fargo, 1st of July in Prosper) due to the warm weather in May and June. However, the flowering period was longer in 2016 (35 days in Fargo, 34 days in Prosper) than in 2015, when the flowering started later (3rd of July in Fargo, 13th of July in Prosper) and took a shorter period (26 in Fargo and 24 days in Prosper) (Table 1 and Supplementary Table S3). Analyzing the DTF data separately for hexaploid lines and tetraploid parents showed that the two groups started flowering about the same time and flowering lasted for about the same period in both years at both locations (data not shown).

The Pearson’s correlation coefficients between FHB severity and PH were significant in the field experiments (*r* = -0.273 and -0.226, *P* < 0.001), indicating that the shorter plants had higher disease severities (Supplementary Table S4). However, no significant correlation was detected between the field PH data and the greenhouse FHB data (*r* = 0.072; *P* = 0.282). Also, the plants that flowered later showed lower disease severity in the field experiments, except in the experiment at the Fargo location in 2016. The PH and DTF did not influence the FHB severity in the greenhouse experiments. Significant correlations were detected among DTF and FHB data collected in various environmental conditions (Supplementary Table S4).

### Decreases of FHB Disease Severities in SHW Lines Compared With Their Tetraploid Parents

Correlation analysis of the FHB severities between SHW lines and their tetraploid parents showed that both the overall data set and the 2016 field data of tetraploids were significantly correlated with the FHB severity data of their SHW lines in all environments (*P* < 0.05) (Table 3). The FHB severity of SHW lines in the greenhouse (SHWGH) was significantly correlated with the data of tetraploids in all environments except in 2015 (Tetr15), suggesting that FHB resistance in the tetraploid parents can be expressed at the hexaploid level.

**Table 3:** Pair-wise correlation coefficients between synthetic hexaploid wheat (SHW) lines and their tetraploid parents for Fusarium head blight (FHB) severity.

Data set	SHW15	SHW16	SHWGH	SHWALL	Tetr15	Tetr16	TetrGH
SHW16	0.138						
SHWGH	0.383^***^	0.363^***^					
SHWALL	0.605^***^	0.795^***^	0.758^***^				
Tetr15	0.098	0.060	0.103	0.113			
Tetr16	0.289^***^	0.412^***^	0.348^***^	0.490^***^	0.547^∗∗∗^		
TetrGH	0.032	-0.039	0.263^**^	0.092	0.505^∗∗∗^	0.507^∗∗∗^	
TetrALL	0.183^*^	0.180^*^	0.290^***^	0.290^***^	0.815^∗∗∗^	0.842^∗∗∗^	0.815^∗∗∗^

*^∗^, ^∗∗^, and ^∗∗∗^ indicate significant at 0.05, 0.01, and 0.001 probability levels, respectively. Data set: SHW15 and SHW16 are combined FHB severity data of SHW lines from both experimental locations (Fargo, Prosper) in 2015 and 2016, respectively; SHWGH and TetrGH are combined FHB severity data of SHW and tetraploid lines, respectively, from the two greenhouse experiments; SHWALL and TetrALL are combined overall FHB severity data of SHW and tetraploid lines, respectively; Tetr15 and Tetr16 are combined FHB severity data of tetraploid lines from both experimental locations (Fargo, Prosper) in 2015 and 2016, respectively.*

A comparison of FHB severities between individual SHW lines and their respective tetraploid parents showed that most SHW lines had lower FHB disease severities than their tetraploid wheat parents, especially under environments with high disease pressures (Supplementary Table S2). The total numbers of the SHW lines with lower FHB disease severities than their tetraploid wheat parents were 80 (55.2%), 135 (91.2%), and 134 (90.5%) in FHB15, FHB16, and FHBGH, respectively. The total numbers of the SHW lines with significant FHB reduction (*P* < 0.05) over their tetraploid wheat parents were 24 (16.6%), 108 (73.0%), and 98 (66.2%) in FHB15, FHB16, and FHBGH, respectively. On the contrary, there were only 14 (9.7%), 1 (0.7%), and 1 (0.7%) SHW lines having significant increases (*P* < 0.05) of FHB severities over their tetraploid parents in FHB15, FHB16, and FHBGH, respectively. Because most of these SHW lines were derived from FHB-susceptible tetraploid parents, the significantly higher FHB severities of these SHW lines were mainly caused by unusually low FHB severities of their tetraploid parents. For example, *T. carthlicum* PI 94751 had FHB severities 72.6% in FHB16 and 56.8% in FHBGH, however, it had FHB severity only 13.7% in FHB15. An FHB-susceptible genotype can occasionally exhibit a resistant reaction with low FHB severity, which might result from unfavorable environmental conditions for disease development or escape of inoculation. This phenomenon commonly occurs in the field FHB evaluation, especially in highly variable weather conditions.

To analyze the effects of the tetraploid subspecies and *Ae. tauschii* genotypes on the FHB resistance of the SHW lines, the percentages of FHB severity reductions in the 140 SHW lines derived from three *Ae. tauschii* accessions (CIae 26, PI 268210, and RL 5286) were grouped by their tetraploid subspecies and *Ae. tauschii* accessions (Table 4). The high levels of FHB severity reductions were largely observed in the SHW lines derived from *T. durum* (27.6%), *T. polonicum* (55.5%), *T. turgidum* (45.2%), and *T. turanicum* (51.4%), whereas low levels of FHB severity reductions were observed in the SHW lines derived from *T. dicoccum* (16.0%) and *T. carthlicum* (11.0%). Similarly, different *Ae. tauschii* genotypes also affected the FHB severities of the SHW lines. Across the six tetraploid subspecies, the three *Ae. tauschii* accessions CIae 26, PI 268210, and RL 5286 resulted in 21.7%, 17.3%, and 11.5% of the FHB severity reduction in their SHW lines, respectively (Table 4). We observed that the highest levels of FHB severity reduction occurred in the SHW lines from the hybrids of *T. turanicum* accessions crossed with *Ae. tauschii* PI 268210 (69.0%) and CIae 26 (67.4%), and *T. polonicum* (66.7%) and *T. durum* (65.1%) accessions crossed with *Ae. tauschii* CIae 26 (data not shown). For all the SHW lines, there was an overall average of 18.3% FHB severity reduction compared with their tetraploid wheat parents (Table 4), indicating that the D genome may play an important role in FHB resistance in wheat.

**Table 4:** Average reductions in Fusarium head blight (FHB) severity calculated from 140 synthetic hexaploid wheat (SHW) lines derived from crosses of six tetraploid wheat subspecies (*T*. *turgidum* ssp.) with three *Ae*. *tauschii* accessions (CIae 26, PI 268210, and RL 5286).

Tetraploid subspecies	CIae 26				PI 268210				RL 5286				Overall Avg
	2015	2016	GH	Avg	2015	2016	GH	Avg	2015	2016	GH	Avg	
*T. durum*	-29.7	-56.5	-56.0	-49.6	-3.8	-34.4	-44.6	-25.9	7.0	-21.6	-9.8	-8.2	-27.6
*T. dicoccum*	-3.5	-25.0	-20.5	-16.3	-5.4	-31.6	-17.9	-18.3	-0.9	-25.5	-10.3	-12.2	-16.0
*T. carthlicum*	6.0	-24.0	-15.9	-11.3	7.2	-23.8	-15.8	-10.8	13.7	-37.3	-9.3	-10.9	-11.0
*T. polonicum*	-47.9	-58.0	-59.6	-55.5									-55.5
*T. turgidum*	-14.5	-50.1	-64.1	-41.3	-24.9	-59.8	-57.7	-44.5					-45.2
*T. turanicum*	-39.0	-63.3	-57.5	-59.2	-49.2	-37.9	-61.4	-51.8					-51.4
Avg	-6.9	-31.1	-26.7	-21.7	-2.6	-29.9	-19.6	-17.3	2.3	-26.9	-10.1	-11.5	-18.3

*2015: Average FHB severity decrease in field experiments (Fargo and Prosper, ND, United States) in 2015. 2016: Average FHB severity decrease in field experiments (Fargo and Prosper, ND, United States) in 2016. GH: Average FHB severity decrease in two greenhouse experiments. Avg: Data were calculated from the average FHB severity data of the individual lines in each environment.*

## Discussion

Synthetic hexaploid wheat has been considered as a valuable germplasm resource for introducing unique genes of agronomically important traits into bread wheat from its closely related or progenitor species in the primary gene pool (Ogbonnaya et al., 2013). For resistance to FHB, Mujeeb-Kazi et al. (2001a,b) evaluated a large number of the SHW lines targeting *Ae. tauschii* genetic diversity developed at CIMMYT and identified 16 SHW lines having a level of resistance as good as the resistant check Sumai 3. Mujeeb-Kazi et al. (2001a) incorporated the FHB-resistant SHW lines into wheat breeding at CIMMYT. Our present study reveals that the SHW lines we recently developed and investigated in our program are also good sources of FHB resistance. Among 149 SHW lines evaluated, many lines showed a high level of FHB resistance in different experiments with 13 lines, namely SW53, SW87, SW91, SW92, SW93, SW157, SW159, SW162, SW188, SW203, SW252, SW253, and SW261, showing FHB severity comparable to the level of Sumai 3 in all experiments. Some of these lines should serve as useful genetic stocks that can be used for development of adapted wheat germplasm and varieties in breeding programs.

It is well known that cultivated tetraploid wheat is more susceptible to FHB than hexaploid wheat (Stack et al., 2002; Oliver et al., 2008; Zhang et al., 2014). As expected, most of the SHW lines evaluated in our study were more resistant than their tetraploid wheat parents in all environments (Supplementary Table S2). On average, 140 SHW lines derived from three *Ae. tauschii* accessions (CIae 26, PI 268210, and RL 5286) decreased their disease severities by 18.3%, suggesting that either the D genome or the increased ploidy level reduced the disease severity in the SHW lines. The data from our experiment provide some evidence to support the hypothesis (Fakhfakh et al., 2011) that the D genome may play an important role in FHB resistance. Conceivably, the D genome may be necessary for expression or increased expression of some FHB resistance QTL located on the A- and/or B-genome chromosomes. It is also possible that the silencing of suppressors present on the A and/or B genome of tetraploids by D genome may lead to expression of resistance.

The evaluation data showed that the FHB severities of the SHW lines varied greatly with different *Ae. tauschii* and tetraploid wheat genotypes involved. The three *Ae. tauschii* accessions, CIae 26, PI 268210, and RL 5286, resulted in 21.7%, 17.3%, and 11.5% of the FHB severity reduction in their SHW lines, respectively (Table 4). Because *Ae. tauschii* CIae 26 and PI 268210 caused the large reduction of FHB severities, they may carry FHB resistance QTL. Therefore, we inferred that the increased FHB resistance in the SHW lines derived from CIae 26 and PI 268210 might be the result of mutual or additive effects from the D genome and its FHB resistance QTL. The two *Ae. tauschii* accessions may have different QTL because they had different effects on the FHB severity reduction in their SHW lines. Brisco et al. (2017) recently evaluated 109 *Ae. tauschii* accessions in the greenhouse and detected significant variation in FHB severity. Among the 10 *Ae. tauschii* accessions in the present study, two accessions, namely CIae 25 (TA1703) and TA 2377, were evaluated for FHB resistance by Brisco et al. (2017) and they were identified as moderately susceptible-moderately resistant and susceptible, respectively. However, in our experiment, their SHW lines (SW8 and SW62) had significant reductions in FHB severities over their durum parent Langdon in two (FHB16 and FHBGH) and three environments, respectively. Because the *Ae. tauschii* parents were not evaluated in our study, we cannot determine if the FHB severities of the SHW lines were associated with those of their *Ae. tauschii* parents. Therefore, further studies are needed to elucidate the relationships of FHB resistance between SHW lines and their *Ae. tauschii* parents by evaluating the SHW lines along with their *Ae. tauschii* parents.

Regarding the effect of tetraploid wheat genotypes on the FHB resistance of the SHW lines, we found that there were positive correlations between the tetraploids and their SHW lines under the environments with high FHB disease pressures in the field nurseries in 2016 (*r* = 0.412, *p* < 0.001) and greenhouse (*r* = 0.263, *p* < 0.01) (Table 3). Most of the SHW lines with a high level of FHB resistance were derived from tetraploid wheat accessions with a high level of FHB resistance. For example, most of *T. polonicum*, *T. turgidum*, and *T. turanicum* accessions evaluated in our study had high disease severities 83.2%, 78.3%, 81.5%, respectively, whereas most *T. dicoccum* and *T. carthlicum* had relatively low disease severities (Supplementary Table S2). The SHW lines derived from these *T. polonicum*, *T. turgidum* and *T. turanicum* accessions had high levels of reductions in FHB severity (55.5%, 45.2%, 51.4%, respectively), whereas those SHW lines derived from the *T. dicoccum* and *T. carthlicum* had low levels of reductions in FHB severity (16.0% and 11.0%, respectively) (Table 4). Noticeably, two *T. dicoccum* accessions PI 272527 and PI 191091 exhibited high levels of FHB resistance in all the environments, suggesting that they may carry major FHB resistance QTL (Table 2). The SHW lines derived from the crosses between these FHB-resistant *T. dicoccum* accessions and different *Ae. tauschii* accessions also consistently showed high levels of FHB resistance across different environments. The lower levels of reductions in FHB severity in the SHW lines involving *T. dicoccum* and *T. carthlicum* is supported by the previous findings that some *T. dicoccum* and *T. carthlicum* accessions have FHB resistance QTL (Gagkaeva, 2003; Clarke et al., 2004; Gladysz et al., 2004; Somers et al., 2006; Buerstmayr et al., 2012). We previously conducted QTL analysis on FHB resistance in two *T. dicoccum* accessions, PI 41025 and PI 272527, and identified two QTL on chromosomes 3A and 5A from PI 41025 and four QTL on chromosomes 1A, 3A, 5A, and 7B, derived from PI 272527 (Zhang et al., 2014, 2017).

In addition to *Ae. tauschii* and tetraploid wheat genotypes, the levels of FHB severity decrease in the SHW lines varied among the environments. On average, there were 29.8% and 21.0% reductions under the environments with high FHB pressures in the field nurseries in 2016 (FHB16) and greenhouse (FHBGH), respectively. However, only a 3.3% reduction was observed under low FHB pressure in the field nurseries in 2015 (FHB15), mostly because the low FHB severities in the field conditions in 2015 reduced the differences between the tetraploids and the SHW lines. This observation is in line with the fact that modern durum crop is more susceptible than bread wheat under the environments with high FHB pressures.

Li et al. (2006) investigated the genetic diversity and genetic relationships among 48 tetraploid wheat accessions belonging to *T*. *turgidum, T*. *durum*, *T*. *carthlicum*, *T*. *paleocolchicum*, *T*. *turanicum*, and *T*. *polonicum* using simple sequence repeat (SSR) markers and grouped *T. durum*, *T. turgidum* and *T. polonicum* into the same cluster in their experiment. Dreisigacker et al. (2008) genotyped a set of 348 accessions from five different tetraploid subspecies using 21 SSR markers and separated *T*. *dicoccum* accessions from *T*. *durum* accessions by principal coordinate analyses. In our study, we found that accessions from *T*. *durum*, *T*. *polonicum*, *T*. *turanicum*, and *T. turgidum* formed a subpopulation, whereas all *T. carthlicum* and most *T. dicoccum* accessions formed two different clusters. Genetic diversity of the SHW lines in our study clearly reflected that of the tetraploid wheat parents. Therefore, these SHW lines represent a unique genetic resource by preserving the high level of genetic diversity from their tetraploid parents.

Lage et al. (2003) analyzed 54 SHW lines derived from 21 *T. dicoccum* and 15 *Ae. tauschii* parental accessions using amplified fragment length polymorphism (AFLP) markers. They also found that the genetic diversity of the SHW lines was associated with the *T. dicoccum* parents rather than their *Ae. tauschii* parents. Dreisigacker et al. (2008) suggested that “SHW diversity would be expected to preferably reflect the diversity of the tetraploid parent” because the tetraploid wheat parent contributed two-thirds of the SHW genome. When genotyping a set of 56 SHW lines derived from durum wheat with only D-genome SSR markers, Dreisigacker et al. (2008) found that the genetic diversity of the SHW lines was closely associated with the subspecies and geographic origin of the *Ae. tauschii* parents. Among the 10 *Ae. tauschii* accessions used in our study, four and six belong to the subspecies *strangulata* and *tauschii*, respectively. The fact that genetic diversity of the SHW lines was not related to the *Ae. tauschii* parents in our study is likely due to the paucity of molecular markers on the D genome because there are approximately ninefold less markers for the D genome than for the A and B genomes.

Use of association mapping analysis to identify FHB resistance genes/QTL in both SHW and tetraploid populations was attempted. However, no associations of markers with significant effects on FHB resistance were detected in either the SHW population or the tetraploid genotypes. This is likely due to the low number of SHW lines used and/or the low frequency of resistance genes and alleles present in the populations. Nonetheless, the results from this study might provide guidance in selecting SHW lines for development of mapping populations to identify FHB resistance genes/QTL using linkage analysis. The SHW lines showing high levels of resistance in all environments, such as SW87, SW162, SW252, SW253, and SW261, might be suitable parents for future development of mapping populations for QTL analysis of FHB resistance.

## Author Contributions

SX and SC initiated and planned this study. AS-H, SC, SX, QZ, SZ, TF, and EE conducted the FHB evaluations in field nurseries and greenhouse. AS-H and SC conducted marker analysis and association mapping. AS-H and SX analyzed the FHB data. SX and YJ conceived and planned the research on development of the SHW lines. QZ, SX, YJ, XC, and JF developed the SHW lines. AS-H, SX, and SC wrote the manuscript. All authors reviewed and edited the manuscript.

## Conflict of Interest Statement

The authors declare that the research was conducted in the absence of any commercial or financial relationships that could be construed as a potential conflict of interest.

## Footnotes

1 https://www.r-project.org/
