# Supplementary material for: Genetic Diversity and Resistance to Fusarium Head Blight in Synthetic Hexaploid Wheat Derived From Aegilops tauschii and Diverse Triticum turgidum Subspecies
Source: Front Plant Sci. 2018 Dec 11;9:1829. doi: 10.3389/fpls.2018.01829 (PMC6298526; doi:10.3389/fpls.2018.01829)
Supplement: Supplementary file 1 [file Table_1.pdf]

Supplementary Table S1 │ Name and origin of the tetraploid wheat (*Ttritcum turgidum*) and *Aegilops tauschii* accessions used as the parents of synthetic hexaploid wheat lines in this study.

Plant ID or Name	Alternate ID or Name	Taxonomy	Origin/Source
Langdon		*T. turgidum* subsp. *durum*	North Dakota, U.S.A.
Ben	PI 596557	*T. turgidum* subsp. *durum*	North Dakota, U.S.A.
Lebsock	PI 613620	*T. turgidum* subsp. *durum*	North Dakota, U.S.A.
8155-B2		*T. turgidum* subsp. *durum*	North Dakota, U.S.A.
Iumillo		*T. turgidum* subsp. *durum*	
CItr 7687	CI 7687	*T. turgidum* subsp. *dicoccom*	Russian Federation
CItr 14133	CI 14133	*T. turgidum* subsp. *dicoccom*	United States
PI 94616	232	*T. turgidum* subsp. *dicoccom*	Russian Federation
PI 94621	238	*T. turgidum* subsp. *dicoccom*	Armenia
PI 94625	242	*T. turgidum* subsp. *dicoccom*	Iran
PI 94626	243	*T. turgidum* subsp. *dicoccom*	Turkey
PI 94627	244	*T. turgidum* subsp. *dicoccom*	Asia Minor
PI 94635	252	*T. turgidum* subsp. *dicoccom*	Iran
PI 94648	265	*T. turgidum* subsp. *dicoccom*	Italy
PI 94666	292	*T. turgidum* subsp. *dicoccom*	Russian Federation
PI 94673	298	*T. turgidum* subsp. *dicoccom*	Armenia
PI 94675	302	*T. turgidum* subsp. *dicoccom*	Georgia
PI 94738	284	*T. turgidum* subsp. *dicoccom*	Ukraine
PI 225332	146	*T. turgidum* subsp. *dicoccom*	Iran
PI 254165	223-a-5	*T. turgidum* subsp. *dicoccom*	Iran
PI 254167	223-a-7	*T. turgidum* subsp. *dicoccom*	Iran
PI 254189	35900	*T. turgidum* subsp. *dicoccom*	Georgia
PI 349043	WIR 6388	*T. turgidum* subsp. *dicoccom*	Georgia
PI 349046	WIR 43848	*T. turgidum* subsp. *dicoccom*	Georgia
PI 352548	T-1520	*T. turgidum* subsp. *dicoccom*	Ethiopia
PI 355507	T 2352	*T. turgidum* subsp. *dicoccom*	Turkey
PI 377655	953	*T. turgidum* subsp. *dicoccom*	Serbia
CItr 3686	CI 3686, Vernal Emmer	*T. turgidum* subsp. *dicoccom*	United States
CItr 7779	CI 7779, 340	*T. turgidum* subsp. *dicoccom*	Ethiopia
CItr 14085	CI 14085	*T. turgidum* subsp. *dicoccom*	Unknown
CItr 14086	CI 14086	*T. turgidum* subsp. *dicoccom*	Unknown
CItr 14135	CI 14135, 2669	*T. turgidum* subsp. *dicoccom*	Ethiopia
PI 41025	859	*T. turgidum* subsp. *dicoccom*	Russian Federation
PI 94618	234	*T. turgidum* subsp. *dicoccom*	Belarus
PI 94669	295	*T. turgidum* subsp. *dicoccom*	Russian Federation
PI 94680	372	*T. turgidum* subsp. *dicoccom*	Germany
PI 94681	373	*T. turgidum* subsp. *dicoccom*	Germany
PI 190926	2475	*T. turgidum* subsp. *dicoccom*	Belgium
PI 191091	Escanda de Malvedo	*T. turgidum* subsp. *dicoccom*	Spain
PI 191390	Rufum	*T. turgidum* subsp. *dicoccom*	Ethiopia
PI 272527	I-1-3428	*T. turgidum* subsp. *dicoccom*	Hungary
PI 61102	Rusak	*T. turgidum* subsp. *carthlicum*	Georgia
PI 78812	CItr 10110	*T. turgidum* subsp. *carthlicum*	Georgia
PI 94748	349	*T. turgidum* subsp. *carthlicum*	Georgia
PI 94750	351	*T. turgidum* subsp. *carthlicum*	Georgia
PI 94751	352	*T. turgidum* subsp. *carthlicum*	Georgia
PI 94752	353	*T. turgidum* subsp. *carthlicum*	Georgia

PI 94753	354	*T. turgidum* subsp. *carthlicum*	Georgia
PI 94754	355	*T. turgidum* subsp. *carthlicum*	Georgia
PI 115816	7106	*T. turgidum* subsp. *carthlicum*	Georgia
PI 283888	Persian	*T. turgidum* subsp. *carthlicum*	Iran
PI 283889	E-682	*T. turgidum* subsp. *carthlicum*	Iran
PI 283890	Persian	*T. turgidum* subsp. *carthlicum*	Iran
PI 352281	T-1819	*T. turgidum* subsp. *carthlicum*	Soviet Union
PI 532489	79TK100-532D-2	*T. turgidum* subsp. *carthlicum*	Turkey
PI 532491	79TK103-544A-2	*T. turgidum* subsp. *carthlicum*	Turkey
PI 532509	H83-1578	*T. turgidum* subsp. *carthlicum*	Canada
PI 532516	H84-562-1	*T. turgidum* subsp. *carthlicum*	United Kingdom
PI 532517	H84-563-2	*T. turgidum* subsp. *carthlicum*	United Kingdom
PI 573182	84TK647-001.1	*T. turgidum* subsp. *carthlicum*	Turkey
PI 585017	AW 6629/85	*T. turgidum* subsp. *carthlicum*	Georgia
Blackbird	REB68421	*T. turgidum* subsp. *carthlicum*	INRA, France
PI 223171	Tafeelih Riti	*T. turgidum* subsp. *polonicum*	Jordan
PI 225335	149	*T. turgidum* subsp. *polonicum*	Iran
PI 254215	46	*T. turgidum* subsp. *polonicum*	Iraq
PI 272567	I-1-3488	*T. turgidum* subsp. *polonicum*	Hungary
PI 272569	I-1-3489	*T. turgidum* subsp. *polonicum*	Hungary
PI 272572	I-1-1769	*T. turgidum* subsp. *polonicum*	Hungary
PI 290512	Gigantil	*T. turgidum* subsp. *polonicum*	Portugal
PI 349051	WIR 39297	*T. turgidum* subsp. *polonicum*	Georgia
PI 349052	WIR 42758	*T. turgidum* subsp. *polonicum*	Azerbaijan
CItr 8115	CI 8115, Sinkiang	*T. turgidum* subsp. *turgidum*	China
CItr 11390	CI 11390, Sun Ray	*T. turgidum* subsp. *turanicum*	United States
PI 185192	Maroccos 24	*T. turgidum* subsp. *turanicum*	Morocco
CIae 17	2134, TA 2463, KU 2080	*Ae. tauschii* subsp. *strangulata*	Mazandaran, Iran
CIae 19	2139	*Ae. tauschii* subsp. *tauschii*	Mazandaran, Iran
CIae 22	2142, TA 2472, KU 2098	*Ae. tauschii* subsp. *tauschii* var. *typica*	Gilan, Iran
CIae 25	2147, TA 1703	*Ae. tauschii* subsp. *tauschii* var. *meyeri*	Gilan, Iran
CIae 26	2152	*Ae. tauschii* subsp. *tauschii*	Gilan, Iran
PI 268210	134, TA 1618	*Ae. tauschii* subsp. *strangulata*	Mazandaran, Iran
PI 476874	WIS 2086, TA1604	*Ae. tauschii* subsp. *tauschii* var. *typica*	Afghanistan
RL 5286		*Ae. tauschii* subsp. *strangulata*	
TA 1675	PI 603236	*Ae. tauschii* subsp. *tauschii*	Turkmenistan
TA 2377	PI 603250	*Ae. tauschii* subsp. *strangulata*	Mazandaran, Iran

[a]CItr, CI, PI, and CIae numbers identifies accessions in USDA National Small Grains Collection, Aberdeen, Idaho, USA. The accession names, pedigrees, growth habits, and sources or origins are specified based on either relevant references or USDA National Plant Germplasm System (http://www.ars-grin.gov/npgs/searchgrin.html). TA number are the accessions at Wheat Genetics Resource Center (WGRC) at Kansas State University in Manhattan, KS. The subspecies of three *Ae. tauschii* accessions (CIae 19, CIae 26, and RL 5286) were designated based on the spike morphology observed in this study and the information of the remaining seven accessions were also provided by Jon Raupp at WGRC.
