# Supplementary material for: Genetic Diversity and Resistance to Fusarium Head Blight in Synthetic Hexaploid Wheat Derived From Aegilops tauschii and Diverse Triticum turgidum Subspecies
Source: Front Plant Sci. 2018 Dec 11;9:1829. doi: 10.3389/fpls.2018.01829 (PMC6298526; doi:10.3389/fpls.2018.01829)
Supplement: Supplementary file 2 [file Table_2.pdf]

Supplementary Table S2 | Fusarium head blight (FHB) severity in the investigated plant material with the rate of reduction in the synthetic hexaploid wheat (SHW) lines. Data were calculated from 2015, 2016, and greenhouse (GH) experiments.

Entry No.	Line	Materials/Pedigree	2015		2016		GH		Overall	
			Avg	Red	Avg	Red	Avg	Red	Avg	Red
**1**	**Langdon**	***T. durum* Langdon**	15.2		53.0		70.1		46.1	
2	SW7	Langdon/CIae 22	14.3	-0.9	23.4**	-29.6	42.0***	-28.1	31.2**	-14.9
3	SW8	Langdon/CIae 25	38.2**	23.0	20.0***	-33.0	47.7**	-22.4	40.0	-6.0
4	SW9	Langdon/CIae 26	n.d.	n.d.	15.0***	-38.0	19.9***	-50.2	18.3***	-27.8
5	SW25	Langdon/RL 5286	29.8	14.7	37.2	-15.8	59.1	-11.0	42.1	-4.0
6	SW52	Langdon/CIae 17	n.d.	n.d.	20.0***	-33.0	49.1**	-21.0	44.2	-1.8
7	SW53	Langdon/PI 268210	11.4	-3.8	18.6***	-34.4	25.5***	-44.6	20.1***	-25.9
8	SW59	Langdon/CIae 19	24.2	9.1	24.8**	-28.2	39.3***	-30.8	31.4**	-14.7
9	SW62	Langdon/TA 2377	25.2	10.0	20.9**	-32.1	52.9*	-17.2	34.8*	-11.2
**10**	**Ben**	***T. durum* Ben**	33.5		69.6		72.6		58.6	
11	SW79	Ben/RL 5286	39.8	6.2	45.7*	-23.9	57.1*	-15.4	47.5*	-11.0
12	SW81	Ben/TA 2377	19.2	-14.4	21.0***	-48.5	46.0***	-26.5	29.7***	-28.9
**13**	**Lebsock**	***T. durum* Lebsock**	33.7		67.0		57.2		51.8	
14	SW84	Lebsock/RL 5286	33.7	0.0	41.9*	-25.1	54.1	-3.1	43.2	-8.6
**15**	**8155-B2**	***T. durum* 8155-B2**	66.9		93.7		89.5		82.8	
16	SW87	8155-B2/CIae 26	20.3***	-46.6	20.8***	-72.9	25.9***	-63.6	22.7***	-60.1
**17**	**Iumillo**	***T. durum* Iumillo**	37.9		84.3		79.3		67.2	
18	SW85	Iumillo/CIae 26	25.2	-12.7	25.6***	-58.6	25.2***	-54.2	25.3***	-41.8
**19**	**CItr 7687-1**	***T. dicoccum* CItr 7687-1**	39.7		98.8		47.8		66.1	
20	SW90	CItr 7687-1/PI 268210	30.6	-9.1	57.1***	-41.7	40.0	-7.8	42.6***	-23.5
**21**	**CItr 14133-1**	***T. dicoccum* CItr 14133-1**	8.1		7.1		42.5		20.6	
22	SW91	CItr 14133-1/CIae 26	9.9	1.8	20.0	12.9	14.7***	-27.8	14.9	-5.8
23	SW92	CItr 14133-1/RL 5286	12.3	4.2	10.3	3.2	17.1***	-25.4	13.4	-7.2
24	SW93	CItr 14133-1/PI 268210	10.5	2.4	25.3	18.2	14.6***	-27.9	16.8	-3.8
**25**	**PI 94616-1**	***T. dicoccum* PI 94616-1**	37.8		79.2		64.4		60.0	
26	SW97	PI 94616-1/CIae 26	20.6*	-17.2	70.5	-8.7	46.8*	-17.5	44.5**	-15.4
27	SW98	PI 94616-1/RL 5286	25.9	-11.9	74.8	-4.4	68.0	3.6	51.4	-8.6
28	SW99	PI 94616-1/PI 268210	22.0	-15.8	72.3	-6.9	56.9	-7.4	49.1*	-10.8
**29**	**PI 94621-1**	***T. dicoccum* PI 94621-1**	43.5		75.8		65.8		61.7	
30	SW100	PI 94621-1/CIae 26	28.7	-14.8	29.6***	-46.2	29.6***	-36.2	29.3***	-32.4

31	SW101	PI 94621-1/RL 5286	36.8	-6.7	30.5***	-45.3	48.4*	-17.3	39.0***	-22.6
32	SW102	PI 94621-1/PI 268210	34.0	-9.5	29.5***	-46.3	41.3***	-24.5	35.2***	-26.4
**33**	**PI 94625-1**	***T. dicoccum* PI 94625-1**	26.8		91.8		59.1		61.1	
34	SW103	PI 94625-1/CIae 26	26.5	-0.3	60.3**	-31.5	29.5***	-29.6	39.5***	-21.6
35	SW104	PI 94625-1/RL 5286	28.6	1.8	69.0*	-22.8	35.9***	-23.2	39.6***	-21.6
36	SW105	PI 94625-1/PI 268210	26.1	-0.7	56.0***	-35.9	39.0**	-20.1	40.3***	-20.8
**37**	**PI 94626-1**	***T. dicoccum* PI 94626-1**	36.8		72.4		55.5		54.9	
38	SW106	PI 94626-1/CIae 26	34.2	-2.7	39.5***	-32.9	35.5**	-19.9	36.4***	-18.5
39	SW107	PI 94626-1/PI 268210	22.7	-14.1	39.8**	-32.6	37.7*	-17.8	34.0***	-20.9
**40**	**PI 94627-1**	***T. dicoccum* PI 94627-1**	46.1		94.9		75.6		72.0	
41	SW108	PI 94627-1/CIae 26	15.9***	-30.2	87.5	-7.5	37.6***	-38.0	47.0***	-25.0
42	SW109	PI 94627-1/RL 5286	39.4	-6.6	59.3***	-35.6	61.1*	-14.6	52.5***	-19.5
43	SW110	PI 94627-1/PI 268210	16.5***	-29.6	87.8	-7.2	68.1	-7.5	54.9***	-17.1
**44**	**PI 94635-1**	***T. dicoccum* PI 94635-1**	25.5		64.6		60.4		50.8	
45	SW111	PI 94635-1/CIae 26	19.1	-6.4	37.0**	-27.5	32.1***	-28.3	32.3***	-18.5
46	SW112	PI 94635-1/PI 268210	21.0	-4.5	42.3*	-22.2	23.6***	-36.9	27.5***	-23.4
**47**	**PI 94648-1**	***T. dicoccum* PI 94648-1**	17.2		84.9		36.3		46.7	
48	SW114	PI 94648-1/CIae 26	30.5	13.4	54.5**	-30.5	28.5	-7.8	36.8*	-9.9
49	SW115	PI 94648-1/PI 268210	41.1**	24.0	54.1**	-30.8	37.5	1.2	44.0	-2.7
**50**	**PI 94666-1**	***T. dicoccum* PI 94666-1**	27.5		81.9		65.8		58.4	
51	SW116	PI 94666-1/CIae 26	25.3	-2.2	32.3***	-49.6	32.4***	-33.3	30.0***	-28.4
52	SW117	PI 94666-1/PI 268210	27.7	0.2	30.7***	-51.2	31.6***	-34.1	30.2***	-28.3
**53**	**PI 94673-1**	***T. dicoccum* PI 94673-1**	38.3		61.0		57.5		51.8	
54	SW118	PI 94673-1/CIae 26	26.8	-11.5	35.7*	-25.3	17.6***	-39.9	26.2***	-25.6
55	SW119	PI 94673-1/RL 5286	43.4	5.1	31.5**	-29.5	31.5***	-26.0	35.5***	-16.3
56	SW120	PI 94673-1/PI 268210	22.3*	-16.0	33.7**	-27.3	24.6***	-32.8	27.2***	-24.6
**57**	**PI 94675-1**	***T. dicoccum* PI 94675-1**	29.5		63.2		49.0		46.3	
58	SW121	PI 94675-1/CIae 26	23.2	-6.3	33.2**	-30.1	25.3***	-23.7	27.2***	-19.1
59	SW122	PI 94675-1/PI 268210	25.8	-3.7	21.3***	-41.9	31.0*	-18.0	26.1***	-20.2
**60**	**PI 94738-1**	***T. dicoccum* PI 94738-1**	23.2		74.1		41.6		44.6	
61	SW123	PI 94738-1/CIae 26	36.3	13.1	36.2***	-37.9	24.2*	-17.3	32.2**	-12.4
62	SW124	PI 94738-1/PI 268210	28.3	5.1	26.4***	-47.7	35.8	-5.7	30.2**	-14.5
**63**	**PI 225332-1**	***T. dicoccum* PI 225332-1**	42.8		78.6		48.5		57.1	
64	SW125	PI 225332-1/CIae 26	30.6	-12.1	59.9	-18.7	28.3**	-20.2	38.9***	-18.2

65	SW126	PI 225332-1/RL 5286	37.8	-5.0	34.0***	-44.6	39.9	-8.6	37.6***	-19.5
66	SW127	PI 225332-1/PI 268210	29.0	-13.7	45.2***	-33.4	35.3	-13.3	36.9***	-20.2
**67**	**PI 254165-1**	***T. dicoccum* PI 254165-1**	33.0		82.1		54.9		56.8	
68	SW128	PI 254165-1/CIae 26	46.6	13.6	72.2	-9.9	35.0**	-19.9	51.3	-5.5
69	SW129	PI 254165-1/RL 5286	39.8	6.8	51.3**	-30.8	38.6*	-16.3	43.2**	-13.5
70	SW130	PI 254165-1/PI 268210	32.5	-0.5	78.5	-3.6	39.2*	-15.7	48.4	-8.4
**71**	**PI 254167-1**	***T. dicoccum* PI 254167-1**	37.8		85.9		69.7		64.8	
72	SW131	PI 254167-1/CIae 26	39.9	2.1	60.0**	-25.9	36.3***	-33.4	45.7***	-19.1
73	SW132	PI 254167-1/RL 5286	30.4	-7.4	44.6***	-41.3	40.0***	-29.7	38.3***	-26.5
**74**	**PI 254189-1**	***T. dicoccum* PI 254189-1**	30.4		85.3		52.1		54.3	
75	SW133	PI 254189-1/CIae 26	35.8	5.4	36.2***	-49.1	37.5*	-14.6	36.5***	-17.8
76	SW134	PI 254189-1/RL 5286	35.1	4.8	43.3***	-41.9	57.8	5.7	45.4	-8.9
77	SW135	PI 254189-1/PI 268210	40.0	9.6	46.1***	-39.1	35.6*	-16.6	40.6**	-13.8
**78**	**PI 349043-1**	***T. dicoccum* PI 349043-1**	37.4		84.5		48.6		57.6	
79	SW138	PI 349043-1/CIae 26	30.5	-6.9	31.5***	-53.0	38.0	-10.5	33.5***	-24.1
80	SW139	PI 349043-1/PI 268210	35.7	-1.7	22.2***	-62.3	27.3**	-21.3	28.7***	-28.8
**81**	**PI 349046-1**	***T. dicoccum* PI 349046-1**	30.6		64.3		52.9		50.3	
82	SW140	PI 349046-1/CIae 26	29.0	-1.6	24.0***	-40.2	32.0**	-20.9	28.3***	-22.0
83	SW141	PI 349046-1/RL 5286	29.2	-1.4	31.2***	-33.1	42.7	-10.2	34.6***	-15.8
84	SW142	PI 349046-1/PI 268210	35.4	4.8	27.2***	-37.0	34.6**	-18.3	32.4***	-17.9
**85**	**PI 352548-1**	***T. dicoccum* PI 352548-1**	47.8		30.9		38.8		39.2	
86	SW143	PI 352548-1/CIae 26	26.6**	-21.2	25.4	-5.5	22.7*	-16.1	24.9**	-14.3
87	SW144	PI 352548-1/RL 5286	26.3**	-21.6	21.1	-9.8	40.7	1.9	29.3*	-9.8
**88**	**PI 355507-1**	***T. dicoccum* PI 355507-1**	40.8		77.5		47.9		55.8	
89	SW145	PI 355507-1/CIae 26	35.8	-5.0	79.0	1.5	35.4	-12.6	50.0	-5.8
90	SW146	PI 355507-1/RL 5286	33.1	-7.6	36.5***	-41.0	43.4	-4.5	37.7***	-18.2
**91**	**PI 377655-1**	***T. dicoccum* PI 377655-1**	26.5		83.2		36.2		50.2	
92	SW147	PI 377655-1/CIae 26	26.0	-0.5	70.5	-12.7	34.0	-2.1	43.5	-6.7
93	SW148	PI 377655-1/PI 268210	14.2	-12.3	85.7	2.6	34.0	-2.2	48.1	-2.1
**94**	**CI 3686**	***T. dicoccum* CI 3686**	37.0		78.5		45.4		53.6	
95	SW150	CI 3686/RL 5286	32.2	-4.9	34.7***	-43.8	43.3	-2.1	36.7***	-16.9
96	SW151	CI 3686/PI 268210	31.7	-5.3	32.8***	-45.7	39.0	-6.4	34.6***	-19.0
**97**	**CI 7779**	***T. dicoccum* CI 7779**	45.1		85.4		44.8		58.5	
98	SW153	CI 7779/CIae 26	27.5*	-17.7	37.0***	-48.4	31.6	-13.2	32.6***	-25.9

99	SW154	CI 7779/PI 268210	29.7	-15.4	37.0***	-48.4	45.6	0.7	38.0***	-20.5
**100**	**CI 14085**	**_T. dicoccum_ CI 14085**	22.1		66.7		61.3		49.1	
101	SW155	CI 14085/CIae 26	35.0	12.9	30.8***	-35.9	24.1***	-37.2	29.7***	-19.4
102	SW156	CI 14085/PI 268210	25.6	3.5	18.8***	-48.0	23.4***	-38.0	22.6***	-26.5
**103**	**CI 14086**	**_T. dicoccum_ CI 14086**	12.6		57.1		54.9		41.5	
104	SW157	CI 14086/CIae 26	20.9	8.2	18.5***	-38.6	25.8***	-29.1	21.9***	-19.7
105	SW158	CI 14086/PI 268210	25.3	12.7	23.0***	-34.1	26.6***	-28.3	24.9***	-16.6
**106**	**CI 14135**	**_T. dicoccum_ CI 14135**	8.5		36.0		24.4		22.9	
107	SW159	CI 14135/CIae 26	21.5	13.1	23.3	-12.8	21.5	-2.9	22.1	-0.8
108	SW160	CI 14135/RL 5286	17.7	9.2	42.3	6.3	29.8	5.4	32.6*	9.7
**109**	**PI 41025**	**_T. dicoccum_ PI 41025**	50.1		73.1		33.1		52.1	
110	SW162	PI 41025/CIae 26	21.3***	-28.8	23.5***	-49.7	16.2*	-16.9	20.3***	-31.8
111	SW163	PI 41025/PI 268210	19.9***	-30.3	32.6***	-40.6	19.7	-13.4	24.5***	-27.6
112	SW164	PI 41025/TA 1675	29.0**	-21.1	46.8**	-26.4	24.4	-8.7	33.4***	-18.7
**113**	**PI 94618**	**_T. dicoccum_ PI 94618**	34.8		89.7		46.6		57.0	
114	SW167	PI 94618/CIae 26	34.5	-0.3	30.5***	-59.2	28.9*	-17.7	31.1***	-25.9
115	SW168	PI 94618/PI 268210	30.6	-4.2	38.3***	-51.4	34.4	-12.2	34.6***	-22.4
**116**	**PI 94669**	**_T. dicoccum_ PI 94669**	28.0		69.7		63.2		51.6	
117	SW171	PI 94669/CIae 26	21.4	-6.6	31.6***	-38.1	29.2***	-33.9	27.2***	-24.4
118	SW172	PI 94669/RL 5286	33.6	5.6	29.5***	-40.2	38.8***	-24.4	34.3***	-17.3
119	SW173	PI 94669/PI 268210	17.3	-10.7	34.0***	-35.7	30.2***	-33.0	29.2***	-22.5
**120**	**PI 94680**	**_T. dicoccum_ PI 94680**	5.0		43.1		48.5		31.6	
121	SW176	PI 94680/RL 5286	25.9**	20.9	26.1	-17.0	27.2**	-21.3	26.4	-5.1
**122**	**PI 94681**	**_T. dicoccum_ PI 94681**	53.6		61.7		52.0		55.7	
123	SW177	PI 94681/CIae 26	32.4**	-21.1	32.6**	-29.1	24.3***	-27.7	29.6***	-26.1
124	SW178	PI 94681/RL 5286	34.9*	-18.7	32.2**	-29.5	42.2	-9.8	36.7***	-19.1
125	SW179	PI 94681/PI 268210	27.7**	-25.8	35.8**	-25.9	28.4***	-23.6	30.5***	-25.2
**126**	**PI 190926**	**_T. dicoccum_ PI 190926**	5.5		42.5		40.1		30.9	
127	SW182	PI 190926/CIae 26	13.6	8.1	33.2	-9.2	17.1**	-23.0	20.8*	-10.1
**128**	**PI 191091**	**_T. dicoccum_ PI 191091**	22.5		28.1		9.3		22.0	
129	SW183	PI 191091/CIae 26	13.1	-9.3	38.7	10.7	15.2	5.9	22.9	0.9
**130**	**PI 191390**	**_T. dicoccum_ PI 191390**	4.9		41.3		40.3		28.2	
131	SW185	PI 191390/CIae 26	15.5	10.6	35.1	-6.2	19.7**	-20.6	21.5	-6.7
132	SW186	PI 191390/PI 268210	15.4	10.5	32.6	-8.7	20.9**	-19.4	21.8	-6.4

**133**	**PI 272527**	***T. dicoccum* PI 272527**	3.4		9.8		8.5		7.2	
134	SW187	PI 272527/CIae 26	9.5	6.0	29.4*	19.6	17.8	9.3	19.6**	12.4
135	SW188	PI 272527/RL 5286	20.5*	17.0	26.5	16.7	30.3**	21.8	25.7***	18.5
**136**	**PI 61102**	***T. carthlicum* PI 61102**	29.3		73.9		38.7		47.3	
137	SW190	PI 61102/CIae 26	42.1	12.8	40.4***	-33.5	34.5	-4.2	39.0	-8.3
138	SW191	PI 61102/PI 268210	32.8	3.5	39.4***	-34.5	37.2	-1.5	36.5*	-10.8
**139**	**PI 78812**	***T. carthlicum* PI 78812**	29.8		74.3		49.4		51.2	
140	SW192	PI 78812 /CIae 26	37.1	7.4	55.2	-19.2	35.5*	-13.9	42.1	-9.0
**141**	**PI 94748**	***T. carthlicum* PI 94748**	20.5		87.0		58.5		55.3	
142	SW193	PI 94748/CIae 26	14.9	-5.6	34.4***	-52.6	32.3***	-26.1	27.5***	-27.8
143	SW194	PI 94748/PI 268210	12.8	-7.6	42.0***	-45.1	27.2***	-31.3	27.3***	-28.0
**144**	**PI 94750**	***T. carthlicum* PI 94750**	33.5		75.7		52.8		54.0	
145	SW195	PI 94750/CIae 26	27.3	-6.2	44.0**	-31.6	36.6*	-16.3	36.0***	-18.0
**146**	**PI 94751**	***T. carthlicum* PI 94751**	13.7		72.6		56.8		47.7	
147	SW197	PI 94751/CIae 26	28.9	15.2	61.3	-11.3	34.0**	-22.8	41.4	-6.3
148	SW198	PI 94751/RL 5286	44.2***	30.5	36.9***	-35.7	48.3	-8.5	43.1	-4.6
149	SW199	PI 94751/PI 268210	27.2	13.5	38.9***	-33.7	29.4***	-27.3	31.8***	-15.9
**150**	**PI 94752**	***T. carthlicum* PI 94752**	19.3		63.3		48.5		43.7	
151	SW200	PI 94752/CIae 26	40.5**	21.2	40.1*	-23.2	33.4*	-15.2	37.8	-5.9
152	SW201	PI 94752/PI 268210	55.2***	36.0	45.6	-17.7	35.7	-12.8	45.5	1.8
**153**	**PI 94753**	***T. carthlicum* PI 94753**	9.9		51.4		37.8		32.0	
154	SW203	PI 94753/PI 268210	10.0	0.1	27.6*	-23.8	30.5	-7.3	22.4*	-9.6
**155**	**PI 94754**	***T. carthlicum* PI 94754**	33.1		42.8		52.6		42.8	
156	SW204	PI 94754/CIae 26	47.5	14.4	32.1	-10.8	36.3*	-16.3	38.6	-4.2
157	SW205	PI 94754/PI 268210	38.2	5.1	41.8	-1.1	29.5***	-23.1	36.3	-6.6
**158**	**PI 115816**	***T. carthlicum* PI 115816**	22.8		72.0		53.0		47.9	
159	SW207	PI 115816/CIae 26	41.9*	19.1	44.6**	-27.5	38.4*	-14.7	41.4	-6.5
160	SW208	PI 115816/RL 5286	32.6	9.9	32.8***	-39.2	48.3	-4.8	37.9*	-10.0
161	SW209	PI 115816/PI 268210	38.8*	16.1	37.6***	-34.5	38.4*	-14.6	38.3*	-9.6
**162**	**PI 283888**	***T. carthlicum* PI 283888**	24.0		67.5		48.6		46.7	
163	SW214	PI 283888/CIae 26	22.3	-1.7	51.1	-16.4	26.3**	-22.3	33.2**	-13.5
164	SW215	PI 283888/PI 476874	31.4	7.4	68.1	0.6	55.3	6.7	51.6	4.9
165	SW216	PI 283888/CIae 22	32.2	8.2	39.3**	-28.2	33.9*	-14.7	35.1*	-11.6
**166**	**PI 283889**	***T. carthlicum* PI 283889**	28.2		87.9		58.6		56.5	

167	SW217	PI 283889/CIae 26	22.4	-5.8	49.5***	-38.3	33.6***	-25.0	35.2***	-21.3
168	SW218	PI 283889/RL 5286	38.8	10.6	48.7***	-39.2	42.1*	-16.5	43.2**	-13.3
**169**	**PI 283890**	***T. carthlicum* PI 283890**	23.6		77.8		41.0		47.5	
170	SW219	PI 283890/CIae 26	26.0	2.5	43.4***	-34.4	28.0	-13.0	32.9**	-14.6
171	SW220	PI 283890/PI 268210	11.6	-11.9	50.5**	-27.3	38.5	-2.5	33.6**	-13.9
**172**	**PI 352281**	***T. carthlicum* PI 352281**	18.7		47.0		52.5		39.4	
173	SW224	PI 352281/CIae 26	15.5	-3.3	66.0	19.0	27.6***	-24.9	37.6	-1.9
174	SW225	PI 352281/PI 268210	27.1	8.3	49.7	2.7	36.9*	-15.6	37.9	-1.5
**175**	**PI 532489**	***T. carthlicum* PI 532489**	25.7		74.6		60.5		53.6	
176	SW226	PI 532489/CIae 26	41.4	15.7	52.7*	-22.0	47.4	-13.2	46.8	-6.8
177	SW227	PI 532489/PI 268210	45.2*	19.5	58.3	-16.3	32.4***	-28.1	45.3	-8.3
**178**	**PI 532491**	***T. carthlicum* PI 532491**	31.0		94.9		62.1		62.7	
179	SW228	PI 532491/CIae 26	48.1*	17.1	59.8***	-35.1	39.2**	-23.0	49.0**	-13.7
**180**	**PI 532509**	***T. carthlicum* PI 532509**	19.7		67.2		45.2		42.7	
181	SW233	PI 532509/CIae 26	38.8*	19.1	42.8*	-24.4	37.4	-7.8	39.7	-3.0
182	SW234	PI 532509/PI 268210	30.6	10.9	32.6***	-34.7	36.6	-8.6	33.3*	-9.4
**183**	**PI 532516**	***T. carthlicum* PI 532516**	25.8		76.5		58.6		52.3	
184	SW236	PI 532516/CIae 26	23.1	-2.7	48.6**	-27.9	28.6***	-30.0	34.1***	-18.2
185	SW237	PI 532516/PI 268210	24.8	-1.0	45.9**	-30.6	31.1***	-27.5	33.3***	-19.0
**186**	**PI 532517**	***T. carthlicum* PI 532517**	19.2		69.3		33.7		40.7	
187	SW238	PI 532517/CIae 26	39.3*	20.0	32.4***	-36.9	34.9	1.2	35.5	-5.2
**188**	**PI 573182**	***T. carthlicum* PI 573182**	29.8		73.0		67.2		56.7	
189	SW242	PI 573182/CIae 26	24.3	-5.6	44.9**	-28.1	45.9**	-21.2	39.2***	-17.5
190	SW243	PI 573182/PI 268210	31.7	1.8	60.8	-12.2	41.1***	-26.0	44.5*	-12.1
**191**	**PI 585017**	***T. carthlicum* PI 585017**	29.8		48.9		29.9		36.2	
192	SW244	PI 585017/CIae 26	21.4	-8.3	43.7	-5.2	36.8	6.9	34.0	-2.2
193	SW245	PI 585017/PI 268210	26.9	-2.9	38.5	-10.4	31.1	1.2	32.2	-4.0
**194**	**Blackbird**	***T. carthlicum* Blackbird**	38.8		65.1		51.1		51.7	
195	SW247	Blackbird/CIae 26	34.4	-4.4	44.6*	-20.5	34.6*	-16.5	37.5**	-14.2
196	SW248	Blackbird/RL 5286	42.8	4.0	30.2***	-34.8	43.9	-7.2	39.0**	-12.7
197	SW249	Blackbird/PI 268210	55.6*	16.8	27.9***	-37.2	40.0	-11.2	41.1*	-10.5
**198**	**PI 223171**	***T. polonicum* PI 223171**	64.2		92.5		93.0		82.7	
199	SW251	PI 223171/CIae 26	30.5***	-33.7	33.4***	-59.1	34.7***	-58.3	32.9***	-49.8
**200**	**PI 225335**	***T. polonicum* PI 225335**	66.3		95.0		97.0		86.1	

201	SW252	PI 225335/CIae 26	15.8***	-50.5	28.3***	-66.7	30.2***	-66.8	25.3***	-60.8
**202**	**PI 254215**	***T. polonicum* PI 254215**	75.9		89.7		95.8		87.2	
203	SW253	PI 254215/CIae 26	19.6***	-56.4	20.0***	-69.8	23.0***	-72.9	20.9***	-66.3
**204**	**PI 272567**	***T. polonicum* PI 272567**	n.d.		84.2		100.0		90.1	
205	SW255	PI 272567/CIae 26	36.6	n.d.	28.2***	-55.9	32.4***	-67.6	32.2***	-57.9
**206**	**PI 272569**	***T. polonicum* PI 272569**	92.8		91.9		91.9		92.1	
207	SW256	PI 272569/CIae 26	19.8***	-73.1	27.4***	-64.5	38.1***	-53.8	27.9***	-64.2
**208**	**PI 272572**	***T. polonicum* PI 272572**	n.d.		n.d.		n.d.		n.d.	
209	SW257	PI 272572/CIae 26	16.7	n.d.	41.0	n.d.	36.7	n.d.	30.3	n.d.
210	SW258	PI 272572/PI 268210	20.9	n.d.	28.1	n.d.	34.1	n.d.	27.7	n.d.
**211**	**PI 290512**	***T. polonicum* PI 290512**	80.6		86.3		87.0		84.9	
212	SW259	PI 290512/CIae 26	31.9***	-48.7	33.9***	-52.4	27.9***	-59.2	31.2***	-53.7
**213**	**PI 349051**	***T. polonicum* PI 349051**	74.8		87.7		65.7		76.1	
214	SW260	PI 349051/CIae 26	20.7***	-54.1	43.6***	-44.0	26.9***	-38.9	30.4***	-45.6
**215**	**PI 349052**	***T. polonicum* PI 349052**	33.6		79.0		77.7		66.4	
216	SW261	PI 349052/CIae 26	15.0*	-18.6	27.7***	-51.2	18.0***	-59.7	20.1***	-46.3
**217**	**CI 8115**	***T. turgidum* CI 8115**	48.1		90.5		99.8		78.3	
218	SW264	CI 8115/CIae 26	33.5	-14.5	40.5***	-50.1	35.7***	-64.1	36.9***	-41.3
219	SW265	CI 8115/PI 268210	23.2**	-24.9	30.7***	-59.8	42.1***	-57.7	33.8***	-44.5
**220**	**CI 11390**	***T. turanicum* CI 11390**	61.3		88.8		95.6		87.8	
221	SW267	CI 11390/CIae 26	22.2***	-39.0	25.5***	-63.3	38.1***	-57.5	28.6***	-59.2
**222**	**PI 185192**	***T. turanicum* PI 185192**	63.8		81.9		79.6		75.1	
223	SW268	PI 185192/PI 268210	14.5***	-49.2	44.0***	-37.9	18.2***	-61.4	23.3***	-51.8
	**Sumai 3**	**Sumai 3**	8.6		9.7		17.1		11.8	
	**Grandin**	**Grandin**	26.4		24.7		55.3		35.5	
Mean			30.1	-3.3	50.4	-29.8	41.7	-21.0	40.8	-17.9
LSD α=0.05			15.8		19.4		13.7		9.4	
LSD α=0.01			20.8		25.6		18.0		12.3	
LSD α=0.001			26.6		32.7		23.0		15.7	

*, **, and *** indicate that the SHW lines were significantly different from their respective tetraploid parents at the 0.05, 0.01, and 0.001 probability levels, respectively (LSD test).

[a]Average (Avg) FHB severity of each genotype from ANOVA (analysis of variance) tests (n.d.: no data).

[b]Reduction (Red) in FHB severity compared to the respective tetraploid parents.

[c]Line number followed by "-1" (e.g. CItr 7687-1) indicated a single plant selection from the original seed stock.
