# Supplementary material for: Genetic Diversity and Resistance to Fusarium Head Blight in Synthetic Hexaploid Wheat Derived From Aegilops tauschii and Diverse Triticum turgidum Subspecies
Source: Front Plant Sci. 2018 Dec 11;9:1829. doi: 10.3389/fpls.2018.01829 (PMC6298526; doi:10.3389/fpls.2018.01829)
Supplement: Supplementary file 3 [file Table_3.pdf]

Supplementary Table S3 | Days to flowering and plant height data (PH) in the investigated plant materials.

Entry			Days to flowering						PH
**No.**	**Accession**	**Subspecies**	15 Pro	15 Far	16 Pro	16 Far	GH	Avg	Avg
**1**	**Langdon**	***T. durum***	31.0	11.3	33.3	35.0	35.2	**30.2**	**120.8**
2	SW7		n.d.	22.5	40.5	40.5	37.7	**36.1**	**97.5**
3	SW8		n.d.	23.3	44.0	45.0	39.0	**35.7**	**96.0**
4	SW9		n.d.	26.0	45.0	38.5	43.8	**41.1**	**100.8**
5	SW25		27.0	10.0	30.7	29.7	31.3	**26.7**	**91.7**
6	SW52		n.d.	n.d.	n.d.	45.0	42.0	**42.5**	**90.0**
7	SW53		n.d.	21.7	43.0	35.0	42.2	**36.5**	**106.4**
8	SW59		n.d.	13.0	38.7	45.0	37.8	**32.8**	**100.0**
9	SW62		34.0	20.0	44.3	35.5	38.8	**35.4**	**108.9**
**10**	**Ben**	***T. durum***	17.0	5.3	21.7	31.3	34.0	**23.9**	**91.7**
11	SW79		24.3	9.7	30.7	30.3	36.7	**28.1**	**90.0**
12	SW81		35.0	17.3	41.3	32.0	44.2	**36.1**	**96.1**
**13**	**Lebsock**	***T. durum***	17.0	8.0	19.3	27.5	28.0	**20.9**	**87.7**
14	SW84		23.0	8.0	26.3	23.3	31.7	**24.0**	**90.0**
**15**	**8155-B2**	***T. durum***	24.7	10.0	31.0	26.5	35.7	**27.3**	**86.4**
16	SW87		35.5	14.0	41.0	43.5	42.3	**37.5**	**83.1**
**17**	**Iumillo**	***T. durum***	23.0	12.3	24.0	25.7	27.3	**23.3**	**95.0**
18	SW85		25.7	13.0	26.7	38.0	31.5	**27.7**	**86.3**
**19**	**CItr 7687-1**	***T. dicoccum***	21.5	9.5	19.0	23.7	30.0	**22.7**	**67.5**
20	SW90		16.7	5.7	22.7	19.0	27.0	**19.7**	**74.1**
**21**	**CItr 14133-1**	***T. dicoccum***	25.7	12.0	25.0	26.7	29.3	**26.3**	**100.0**
22	SW91		21.0	13.7	24.7	24.3	32.3	**24.7**	**92.9**
23	SW92		22.0	15.0	29.0	18.5	29.7	**24.3**	**96.4**
24	SW93		23.0	12.7	28.7	30.3	32.5	**26.6**	**92.5**
**25**	**PI 94616-1**	***T. dicoccum***	16.7	6.3	16.0	17.0	27.3	**17.3**	**79.6**
26	SW97		13.0	3.0	16.0	10.0	20.3	**14.0**	**81.4**
27	SW98		15.7	5.7	19.0	n.d.	31.0	**19.7**	**82.8**
28	SW99		15.0	5.0	15.3	20.5	21.0	**16.1**	**81.8**
**29**	**PI 94621-1**	***T. dicoccum***	21.0	11.0	21.3	21.0	28.8	**22.0**	**85.0**
30	SW100		18.0	8.0	19.0	23.3	28.2	**20.9**	**86.8**
31	SW101		19.3	6.3	19.3	13.5	22.8	**17.6**	**85.5**
32	SW102		20.0	10.7	26.3	19.0	28.7	**22.4**	**81.4**
**33**	**PI 94625-1**	***T. dicoccum***	20.0	7.3	17.3	19.0	28.0	**19.9**	**68.6**
34	SW103		20.7	5.3	18.0	11.0	29.7	**19.1**	**74.1**
35	SW104		18.0	8.0	19.7	n.d.	24.0	**18.7**	**83.9**
36	SW105		23.0	6.3	19.3	15.0	29.0	**20.3**	**75.4**
**37**	**PI 94626-1**	***T. dicoccum***	20.0	15.7	19.3	24.3	36.3	**25.3**	**93.3**
38	SW106		19.0	7.0	20.7	19.0	27.5	**20.1**	**84.2**
39	SW107		25.7	11.0	23.3	15.0	31.0	**25.6**	**87.8**
**40**	**PI 94627-1**	***T. dicoccum***	17.7	8.3	19.7	23.7	24.2	**19.4**	**78.3**
41	SW108		13.0	3.7	18.3	22.7	20.2	**16.3**	**77.9**
42	SW109		15.3	6.0	18.3	13.0	19.8	**15.7**	**84.5**
43	SW110		13.3	4.7	16.7	17.5	20.0	**15.2**	**79.1**

**44**	**PI 94635-1**	***T. dicoccum***	37.0	28.0	38.7	37.0	46.3	**39.0**	**101.1**
45	SW111		25.0	n.d.	45.0	36.5	39.6	**37.9**	**90.8**
46	SW112		n.d.	29.0	n.d.	38.3	44.5	**41.1**	**90.0**
**47**	**PI 94648-1**	***T. dicoccum***	24.3	16.0	27.7	28.0	51.2	**32.0**	**92.1**
48	SW114		25.3	9.7	22.0	28.0	38.3	**26.9**	**86.8**
49	SW115		23.7	11.0	22.0	19.0	32.2	**25.3**	**86.7**
**50**	**PI 94666-1**	***T. dicoccum***	20.0	13.7	23.0	28.3	34.3	**25.6**	**81.3**
51	SW116		21.0	9.0	22.0	14.0	31.7	**21.6**	**77.1**
52	SW117		20.0	15.0	23.0	16.3	34.5	**24.1**	**82.7**
**53**	**PI 94673-1**	***T. dicoccum***	19.0	13.7	27.7	17.5	36.5	**25.6**	**80.0**
54	SW118		25.0	10.3	22.0	18.0	31.0	**23.2**	**80.0**
55	SW119		23.7	8.3	20.3	22.7	23.0	**20.2**	**78.3**
56	SW120		25.7	10.0	22.0	24.0	27.8	**23.6**	**78.6**
**57**	**PI 94675-1**	***T. dicoccum***	22.3	11.3	25.3	13.5	33.7	**23.9**	**99.1**
58	SW121		22.0	8.7	20.3	25.7	28.2	**22.2**	**86.7**
59	SW122		16.3	7.3	25.0	20.7	29.3	**21.3**	**90.4**
**60**	**PI 94738-1**	***T. dicoccum***	19.0	13.7	24.7	18.0	34.8	**24.5**	**99.5**
61	SW123		20.0	8.0	23.3	21.3	27.0	**21.1**	**89.6**
62	SW124		16.0	9.0	22.0	19.0	29.2	**20.7**	**87.9**
**63**	**PI 225332-1**	***T. dicoccum***	17.7	8.0	21.7	24.7	27.4	**20.8**	**82.5**
64	SW125		20.3	9.0	23.3	24.5	22.2	**20.7**	**76.5**
65	SW126		16.0	8.0	24.0	17.0	19.3	**17.3**	**82.0**
66	SW127		19.0	7.5	16.7	17.3	24.5	**18.9**	**78.6**
**67**	**PI 254165-1**	***T. dicoccum***	17.0	5.3	18.7	22.7	33.4	**21.1**	**77.5**
68	SW128		18.0	6.7	20.7	16.7	25.5	**18.8**	**76.3**
69	SW129		16.3	7.7	18.0	17.7	24.5	**18.1**	**76.3**
70	SW130		18.3	8.7	21.3	24.5	27.8	**21.2**	**74.1**
**71**	**PI 254167-1**	***T. dicoccum***	22.0	7.3	17.3	22.5	28.8	**21.0**	**79.5**
72	SW131		16.7	9.0	16.7	19.0	25.2	**19.2**	**75.9**
73	SW132		16.3	5.7	20.7	16.3	21.7	**17.1**	**80.8**
**74**	**PI 254189-1**	***T. dicoccum***	22.0	13.0	26.0	24.0	41.0	**27.3**	**91.4**
75	SW133		22.3	9.0	23.3	28.3	29.8	**23.8**	**82.1**
76	SW134		18.0	11.3	24.7	18.7	27.2	**21.2**	**81.7**
77	SW135		23.7	15.0	23.0	37.0	29.7	**26.3**	**81.3**
**78**	**PI 349043-1**	***T. dicoccum***	22.0	12.0	24.0	24.0	34.8	**26.5**	**93.9**
79	SW138		30.0	10.0	18.7	27.7	29.7	**23.9**	**80.5**
80	SW139		20.3	10.0	25.7	12.0	30.2	**23.4**	**85.6**
**81**	**PI 349046-1**	***T. dicoccum***	18.0	13.5	21.7	22.0	35.0	**24.8**	**90.0**
82	SW140		21.3	11.7	18.7	22.0	30.0	**22.3**	**81.3**
83	SW141		18.7	12.7	19.3	23.0	26.3	**20.9**	**87.3**
84	SW142		20.0	8.7	21.3	16.0	31.3	**21.4**	**87.5**
**85**	**PI 352548-1**	***T. dicoccum***	15.3	4.3	13.7	13.0	20.0	**14.4**	**85.4**
86	SW143		16.7	6.0	20.7	10.5	22.2	**16.7**	**90.0**
87	SW144		16.0	5.7	14.0	11.7	19.0	**14.2**	**85.8**
**88**	**PI 355507-1**	***T. dicoccum***	18.0	8.7	18.0	21.3	27.6	**19.8**	**80.8**
89	SW145		19.0	9.0	19.7	18.0	26.7	**19.8**	**79.6**
90	SW146		18.7	9.3	16.0	14.7	24.5	**17.9**	**83.3**
**91**	**PI 377655-1**	***T. dicoccum***	23.3	13.7	25.0	23.3	44.0	**27.0**	**99.2**

92	SW147		17.0	6.7	20.0	30.0	29.8	**22.2**	**85.8**
93	SW148		18.0	10.5	24.0	31.0	28.3	**23.5**	**85.0**
**94**	**CI 3686**	***T. dicoccum***	20.0	11.7	24.7	25.0	33.2	**24.6**	**99.6**
95	SW150		18.0	8.7	18.7	28.3	24.7	**20.5**	**92.5**
96	SW151		17.0	10.3	23.7	24.0	29.8	**22.4**	**93.2**
**97**	**CI 7779**	***T. dicoccum***	20.0	11.3	25.7	27.0	34.3	**25.4**	**94.6**
98	SW153		21.3	10.0	23.0	21.3	27.8	**23.4**	**87.0**
99	SW154		21.7	12.0	26.7	32.0	27.5	**24.4**	**86.1**
**100**	**CI 14085**	***T. dicoccum***	37.0	25.7	35.7	33.3	34.2	**33.3**	**102.1**
101	SW155		19.0	7.0	19.3	28.0	25.3	**21.5**	**94.1**
102	SW156		21.0	12.3	23.7	28.3	27.0	**23.2**	**95.4**
**103**	**CI 14086**	***T. dicoccum***	36.0	22.7	38.0	38.3	32.5	**33.3**	**104.6**
104	SW157		17.0	10.0	19.3	23.7	25.0	**21.3**	**96.0**
105	SW158		23.3	9.5	20.7	17.7	24.3	**20.6**	**98.2**
**106**	**CI 14135**	***T. dicoccum***	36.0	25.7	34.3	36.7	38.6	**34.8**	**103.8**
107	SW159		23.3	10.0	25.7	21.7	25.3	**22.6**	**92.3**
108	SW160		n.d.	22.0	31.0	31.0	20.8	**24.3**	**73.8**
**109**	**PI 41025**	***T. dicoccum***	20.0	10.3	25.0	26.3	38.3	**26.4**	**105.4**
110	SW162		17.7	7.7	16.0	19.7	24.0	**18.2**	**89.2**
111	SW163		20.5	12.5	20.0	19.0	28.0	**21.9**	**92.0**
112	SW164		18.7	13.0	23.7	30.3	35.2	**26.0**	**82.9**
**113**	**PI 94618**	***T. dicoccum***	20.0	11.7	25.0	26.7	42.2	**27.9**	**93.3**
114	SW167		21.5	8.0	19.3	18.5	31.0	**21.8**	**85.0**
115	SW168		25.0	11.0	23.0	14.3	28.2	**22.2**	**82.3**
**116**	**PI 94669**	***T. dicoccum***	22.0	12.0	24.5	26.5	33.0	**25.1**	**101.5**
117	SW171		19.0	10.0	20.7	20.5	29.0	**21.4**	**89.1**
118	SW172		22.0	9.0	19.3	22.5	24.0	**20.7**	**93.0**
119	SW173		28.0	22.0	25.0	24.3	30.7	**27.3**	**84.4**
**120**	**PI 94680**	***T. dicoccum***	37.0	23.7	41.7	34.0	36.3	**34.8**	**106.8**
121	SW176		22.7	14.3	24.0	17.0	24.0	**21.5**	**94.5**
**122**	**PI 94681**	***T. dicoccum***	17.0	10.0	17.3	23.7	31.7	**21.9**	**95.0**
123	SW177		18.0	7.3	19.7	20.5	27.3	**20.0**	**87.3**
124	SW178		16.3	10.7	21.0	18.5	25.2	**19.5**	**89.1**
125	SW179		17.0	9.0	24.0	19.3	27.7	**21.3**	**87.5**
**126**	**PI 190926**	***T. dicoccum***	37.0	28.0	37.0	41.0	36.5	**36.1**	**105.0**
127	SW182		27.0	14.7	27.7	18.0	30.8	**24.9**	**93.2**
**128**	**PI 191091**	***T. dicoccum***	n.d.	19.3	36.3	35.3	45.0	**34.0**	**107.5**
129	SW183		30.0	14.5	24.3	36.3	37.0	**30.8**	**91.8**
**130**	**PI 191390**	***T. dicoccum***	37.0	27.3	37.7	31.7	42.2	**36.0**	**102.1**
131	SW185		27.0	19.5	28.0	36.0	29.5	**27.8**	**93.8**
132	SW186		27.7	13.3	30.7	25.0	29.0	**25.9**	**96.0**
**133**	**PI 272527**	***T. dicoccum***	37.0	23.3	44.7	40.3	60.5	**40.0**	**130.0**
134	SW187		32.0	19.0	35.7	38.0	39.4	**34.4**	**100.0**
135	SW188		27.0	14.3	33.3	24.5	36.2	**28.8**	**104.1**
**136**	**PI 61102**	***T. carthlicum***	22.0	12.0	25.7	24.3	30.2	**24.1**	**92.5**
137	SW190		19.0	13.0	22.0	21.3	24.8	**20.8**	**89.6**
138	SW191		17.0	9.3	20.7	14.3	23.7	**18.1**	**92.1**
**139**	**PI 78812**	***T. carthlicum***	23.0	9.0	26.0	23.0	31.3	**23.9**	**93.3**

140	SW192		28.3	13.5	28.7	30.0	32.8	**28.4**	**91.0**
**141**	**PI 94748**	***T. carthlicum***	24.0	9.7	22.0	21.0	35.5	**24.6**	**88.3**
142	SW193		35.0	17.0	34.0	36.0	37.8	**33.8**	**89.4**
143	SW194		33.0	20.3	36.3	36.0	39.5	**34.1**	**84.2**
**144**	**PI 94750**	***T. carthlicum***	20.0	8.7	22.0	22.3	33.7	**23.4**	**92.5**
145	SW195		24.3	9.7	28.3	30.7	30.8	**25.8**	**88.3**
**146**	**PI 94751**	***T. carthlicum***	27.7	13.3	22.0	28.0	28.7	**24.7**	**92.9**
147	SW197		29.0	13.3	29.3	28.3	32.2	**27.4**	**89.2**
148	SW198		25.7	13.0	29.0	24.7	28.2	**24.8**	**91.3**
149	SW199		28.3	14.0	32.7	32.3	32.2	**28.6**	**88.3**
**150**	**PI 94752**	***T. carthlicum***	28.3	10.3	23.3	28.0	28.8	**24.6**	**87.9**
151	SW200		20.0	10.0	23.0	18.3	24.2	**20.5**	**90.5**
152	SW201		19.0	9.0	23.0	24.5	25.7	**20.9**	**89.5**
**153**	**PI 94753**	***T. carthlicum***	28.3	13.7	29.3	27.5	37.0	**28.9**	**96.8**
154	SW203		36.0	17.3	40.3	34.0	40.7	**34.9**	**89.0**
**155**	**PI 94754**	***T. carthlicum***	27.0	14.0	25.3	20.0	26.8	**23.3**	**88.8**
156	SW204		21.0	13.0	18.0	19.7	23.2	**19.7**	**89.6**
157	SW205		22.0	10.0	20.7	26.0	24.2	**22.6**	**86.8**
**158**	**PI 115816**	***T. carthlicum***	24.3	12.0	25.0	26.5	28.5	**24.0**	**101.8**
159	SW207		17.3	13.3	17.3	30.0	21.3	**19.5**	**93.2**
160	SW208		17.7	7.7	20.0	24.0	21.5	**18.7**	**94.6**
161	SW209		19.3	9.3	21.3	21.5	22.2	**19.2**	**90.0**
**162**	**PI 283888**	***T. carthlicum***	22.0	9.0	22.0	21.0	33.8	**23.6**	**92.1**
163	SW214		29.7	14.3	30.0	17.7	33.7	**26.5**	**91.3**
164	SW215		20.0	6.7	28.0	19.7	23.8	**20.3**	**86.7**
165	SW216		24.3	11.3	26.7	17.3	29.3	**23.1**	**91.3**
**166**	**PI 283889**	***T. carthlicum***	20.0	8.7	23.7	27.5	34.0	**24.5**	**92.7**
167	SW217		31.3	13.3	28.7	34.0	34.0	**29.2**	**88.8**
168	SW218		22.0	10.7	28.0	20.7	27.0	**22.6**	**91.3**
**169**	**PI 283890**	***T. carthlicum***	21.0	9.7	22.0	22.7	33.3	**23.7**	**90.8**
170	SW219		26.3	13.5	28.3	29.3	31.2	**27.4**	**88.2**
171	SW220		30.7	12.7	30.3	25.7	33.7	**27.8**	**87.5**
**172**	**PI 352281**	***T. carthlicum***	24.3	11.3	24.0	13.7	33.3	**23.3**	**92.9**
173	SW224		28.3	15.5	32.7	26.3	34.0	**29.2**	**89.2**
174	SW225		27.7	10.3	33.0	24.3	32.8	**26.8**	**93.3**
**175**	**PI 532489**	***T. carthlicum***	17.7	8.0	20.0	23.3	28.8	**21.1**	**84.6**
176	SW226		21.0	8.7	23.7	15.5	29.3	**21.6**	**86.4**
177	SW227		27.0	12.3	27.3	29.0	32.8	**26.9**	**87.5**
**178**	**PI 532491**	***T. carthlicum***	17.0	8.0	22.0	34.7	27.7	**22.8**	**82.9**
179	SW228		23.3	10.7	27.3	32.3	31.3	**26.1**	**84.6**
**180**	**PI 532509**	***T. carthlicum***	24.3	12.0	24.3	31.5	27.7	**24.2**	**91.4**
181	SW233		19.0	8.7	20.0	21.0	22.5	**18.9**	**90.4**
182	SW234		17.0	10.3	20.7	13.0	25.3	**18.6**	**90.8**
**183**	**PI 532516**	***T. carthlicum***	21.0	7.3	23.0	31.0	31.3	**24.2**	**91.8**
184	SW236		29.3	10.5	31.0	25.3	31.3	**27.4**	**88.8**
185	SW237		30.7	13.7	34.5	24.0	33.8	**28.1**	**85.5**
**186**	**PI 532517**	***T. carthlicum***	25.7	12.7	27.0	31.3	31.3	**26.6**	**87.9**
187	SW238		17.0	13.0	22.3	13.0	21.8	**18.8**	**84.5**

**188**	**PI 573182**	***T. carthlicum***	22.0	12.7	23.7	16.7	27.7	**21.7**	**88.8**
189	SW242		32.5	13.3	31.7	21.0	31.8	**26.7**	**86.4**
190	SW243		25.7	9.0	30.3	31.7	33.3	**27.2**	**90.8**
**191**	**PI 585017**	***T. carthlicum***	27.7	13.0	28.3	25.3	33.3	**26.8**	**92.5**
192	SW244		35.0	13.0	34.7	28.7	37.5	**31.1**	**90.0**
193	SW245		32.0	16.0	33.0	32.7	41.8	**32.9**	**85.4**
**194**	**Blackbird**	***T. carthlicum***	21.0	11.3	25.3	22.7	25.7	**21.9**	**86.3**
195	SW247		16.7	7.0	21.3	23.0	24.3	**19.4**	**85.0**
196	SW248		18.7	10.0	18.0	19.0	20.0	**17.6**	**86.3**
197	SW249		20.7	8.3	18.0	12.3	23.0	**17.6**	**87.1**
**198**	**PI 223171**	***T. polonicum***	22.0	14.0	26.3	28.0	32.8	**25.9**	**87.7**
199	SW251		27.0	11.0	30.0	34.7	38.8	**30.1**	**88.3**
**200**	**PI 225335**	***T. polonicum***	23.5	13.0	30.0	34.0	28.6	**25.8**	**83.5**
201	SW252		27.0	11.5	27.7	28.3	35.7	**28.6**	**97.7**
**202**	**PI 254215**	***T. polonicum***	22.0	13.3	26.7	37.0	31.0	**26.8**	**90.8**
203	SW253		27.0	14.7	29.7	28.5	31.8	**27.2**	**95.0**
**204**	**PI 272567**	***T. polonicum***	n.d.	n.d.	30.0	18.5	33.0	**28.3**	**76.9**
205	SW255		27.7	14.0	27.3	22.7	36.0	**28.1**	**100.5**
**206**	**PI 272569**	***T. polonicum***	29.0	12.0	31.3	31.0	30.0	**29.1**	**79.4**
207	SW256		27.7	12.7	26.7	32.3	35.2	**28.3**	**100.8**
**208**	**PI 272572**	***T. polonicum***	n.d.	n.d.	n.d.	n.d.	n.d.	**n.d.**	**n.d.**
209	SW257		30.7	12.0	27.0	21.0	36.0	**27.5**	**95.5**
210	SW258		27.0	10.7	28.3	22.5	38.7	**27.9**	**97.3**
**211**	**PI 290512**	***T. polonicum***	25.7	13.5	29.3	31.3	29.5	**27.2**	**82.1**
212	SW259		30.7	17.5	29.7	32.7	34.2	**30.5**	**97.7**
**213**	**PI 349051**	***T. polonicum***	27.0	14.7	30.7	34.3	37.7	**30.3**	**96.7**
214	SW260		29.7	17.7	32.0	28.0	38.8	**30.8**	**104.2**
**215**	**PI 349052**	***T. polonicum***	37.0	22.0	35.7	28.5	47.7	**36.9**	**107.2**
216	SW261		35.5	16.3	35.7	30.5	41.5	**33.6**	**102.5**
**217**	**CI 8115**	***T. turgidum***	25.7	10.0	28.0	20.0	27.2	**22.8**	**110.9**
218	SW264		n.d.	24.0	42.0	36.5	42.2	**38.2**	**92.9**
219	SW265		n.d.	18.0	43.3	38.0	37.3	**34.8**	**97.2**
**220**	**CI 11390**	***T. turanicum***	n.d.	17.0	27.3	25.0	43.0	**32.1**	**95.0**
221	SW267		33.0	16.0	34.3	30.7	45.0	**34.0**	**93.8**
**222**	**PI 185192**	***T. turanicum***	22.0	9.3	23.7	24.7	32.2	**24.0**	**96.7**
223	SW268		33.0	16.7	34.0	26.0	44.7	**34.1**	**90.0**
	**Sumai 3**		19.7	5.0	24.7	11.3	24.8	**18.4**	**85.4**
	**Grandin**		13.3	5.7	11.0	12.7	18.3	**13.2**	**80.4**
Average			23.0	11.8	25.5	25.0	31.1	**24.7**	**89.7**
LSD α=0.05			4.2	5.0	4.6	14.7	4.7	**3.4**	**5.8**

[a]15 Pro and 16 Pro: Days to flowering in Prosper in 2015 and 2016, respectively.
[b]15 Far and 16 Far: Days to flowering in Fargo in 2015 and 2016 respectively.
[c]Average days to flowering from the two greenhouse experiments from ANOVA test.
[d]Average plant height data from all field experiments (2015, 2016) from ANOVA test.
n.d.: no data
