# Supplementary material for: Genetic Diversity and Resistance to Fusarium Head Blight in Synthetic Hexaploid Wheat Derived From Aegilops tauschii and Diverse Triticum turgidum Subspecies
Source: Front Plant Sci. 2018 Dec 11;9:1829. doi: 10.3389/fpls.2018.01829 (PMC6298526; doi:10.3389/fpls.2018.01829)
Supplement: Supplementary file 4 [file Table_4.pdf]

Supplementary Table S4 | Pair-wise correlation coefficients between the investigated traits.

Data set	DTF15 Pro	DTF15 Far	DTF16 Pro	DTF16 Far	DTF GH	PH	FHB15	FHB16
DTF15 Far	0.792***							
DTF16 Pro	0.840***	0.795***						
DTF16 Far	0.623***	0.641***	0.693***					
DTF GH	0.692***	0.662***	0.727***	0.598***				
PH	0.498***	0.539***	0.552***	0.413***	0.523***			
FHB15	-0.338***	-0.294***	-0.282***	-0.158*	-0.255***	-0.273***		
FHB16	-0.173*	-0.192**	-0.209**	-0.030	0.001	-0.226***	0.412***	
FHB GH	-0.109	-0.122	-0.081	0.003	-0.057	-0.072	0.565***	0.685***

*, **, and *** indicate significant at 0.05, 0.01, and 0.001 probability levels, respectively.

[a]Data set: DTF15 Pro and DTF16 Pro are days to flowering in Prosper in 2015 and 2016, respectively; DTF15 Far and DTF16 Far are days to flowering in Fargo in 2015 and 2016, respectively; DTF GH is average days to flowering data from the two greenhouse experiments; PH is average plant height data from all field experiments (2015 and 2016); FHB15 and FHB 16 are average FHB severity data from both experimental locations (Fargo and Prosper) in 2015 and 2016, respectively; FHB GH is average FHB severity data from the two greenhouse experiments.
