# Supplementary material for: Genetic Diversity and Resistance to Fusarium Head Blight in Synthetic Hexaploid Wheat Derived From Aegilops tauschii and Diverse Triticum turgidum Subspecies
Source: Front Plant Sci. 2018 Dec 11;9:1829. doi: 10.3389/fpls.2018.01829 (PMC6298526; doi:10.3389/fpls.2018.01829)
Supplement: Supplementary file 5 [file Presentation_1.pdf]

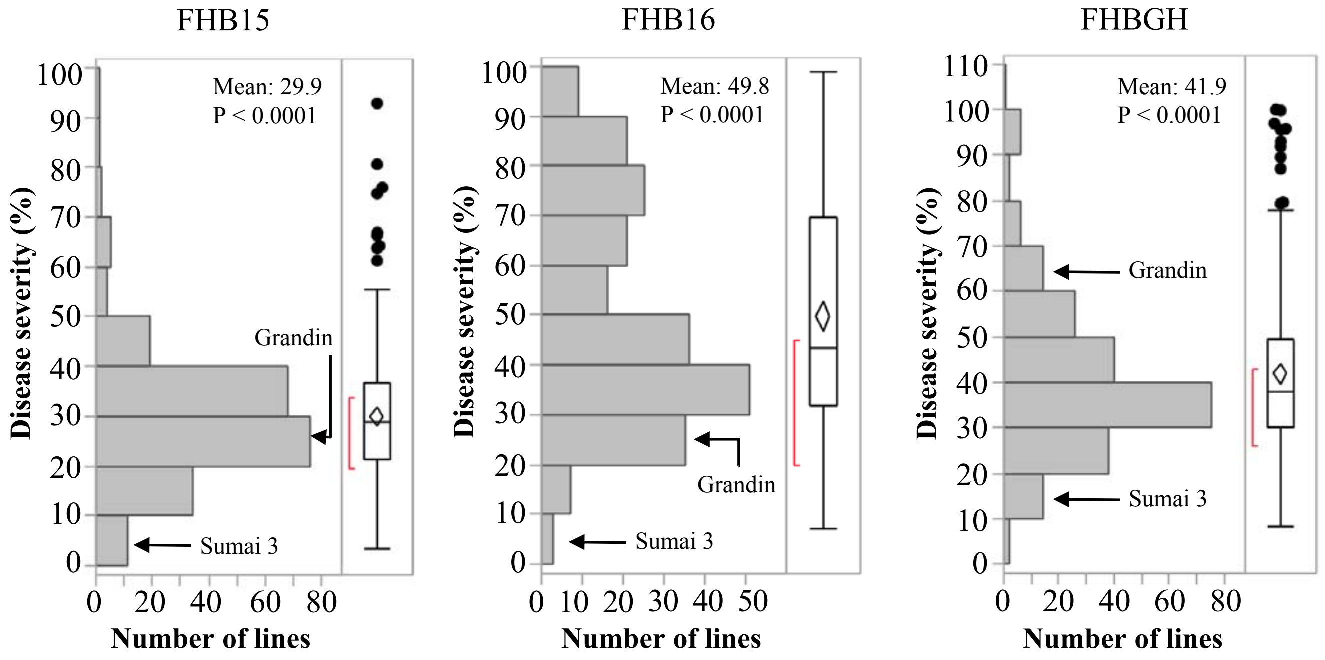

SUPPLEMENTARY FIGURE S1 | Distribution of Fusarium head blight (FHB) disease severity among the SHW lines (n = 149) and tetraploid parents (n = 73) including the two checks, Sumai 3 and Grandin in the three environments. FHB15 and FHB16 are combined FHB severity data from both experimental locations (Fargo and Prosper) in 2015 and 2016, respectively. FHBGH is combined FHB severity data from the two greenhouse experiments. Letter "P" represents probability from normality test for distribution of disease severity.
